# From the Cardiovascular–Kidney–Metabolic Disorders to the Atherosclerotic Cardiovascular Diseases: Their Prevalence Rates and Independent Associations in the SIMETAP Study

**DOI:** 10.3390/jcm14113940

**Published:** 2025-06-03

**Authors:** Antonio Ruiz-García, Vicente Pallarés-Carratalá, Adalberto Serrano-Cumplido, Vicente Pascual-Fuster, Ezequiel Arranz-Martínez, Carlos Escobar-Cervantes

**Affiliations:** 1 Lipids and Cardiovascular Prevention Unit, Pinto University Health Centre, 28320 Madrid, Spain; antoniodoctor@gmail.com; 2 Department of Medicine, European University of Madrid, 28005 Madrid, Spain; 3 Department of Medicine, Jaume I University, 12006 Castellon, Spain; pallarev@uji.es; 4 Repelega Health Centre, 48920 Portugalete, Spain; 5 Palleter Health Centre, 12005 Castellon, Spain; pascual_vic@gva.es; 6 San Blas Health Centre, 28981 Madrid, Spain; ezequielarranz@gmail.com; 7 Department of Cardiology, La Paz University Hospital, 28046 Madrid, Spain

**Keywords:** adults, atherosclerotic cardiovascular disease, cardiovascular–kidney–metabolic syndrome, chronic kidney disease, coronary heart disease, peripheral arterial disease, prevalence, stroke

## Abstract

**Background/Objectives:** Atherosclerotic cardiovascular diseases (ASCVDs) remain the leading cause of morbimortality worldwide. The objectives of this study were to update the prevalence rates of ASCVDs and to evaluate their relationship with cardiovascular–kidney–metabolic (CKM) disorders. **Methods:** This cross-sectional observational study included 6588 adults selected through a simple random population-based sample from the Health Service database of the Madrid Region (Spain). Adjusted prevalence rates were calculated by the direct method, according to Spanish population data from the National Institute of Statistics. The relationships of CKM disorders with coronary heart disease (CHD), stroke, peripheral arterial disease (PAD), and ASCVD were assessed by bivariate and multivariate analyses. **Results:** The age- and sex-adjusted prevalence rates among overall adults with CHD, stroke, PAD, and ASCVD were 3.8%, 3.0%, 1.8%, and 7.3%, respectively, and they reached 5.6%, 4.4%, 2.6%, and 10.8%, respectively, among people aged 40 years and older. The prevalence rates were higher in men than women aged over 40 years for CHD and ASCVD, between 50 and 69 years for stroke, and aged over 60 years for PAD. The mean ages of women and men with ASCVD were 74.9 and 70.2 years, respectively. Hypertension, heart failure (HF), hypercholesterolaemia, diabetes, low eGFR, atrial fibrillation (AF), prediabetes, and low HDL-c were independently associated with ASCVD, highlighting hypertension and HF for all of them, in addition to hypercholesterolaemia for CHD and stroke, and specifically, AF for stroke. **Conclusions:** More than one in ten people aged over 40 suffer from CHD, stroke, or PAD. Hypertension, HF, hypercholesterolaemia, diabetes, and low eGFR are the major CKM disorders associated with ASCVD.

## 1. Introduction

Mortality from cardiovascular diseases (CVDs) remains the leading cause of death worldwide, despite a 34.9% decrease in CVD mortality worldwide between 1990 and 2022, due to both improved care at the time of the atherosclerotic event and subsequent chronic management of atherosclerotic cardiovascular disease (ASCVD) [1,2,3]. Cardiovascular deaths are projected to increase from 20.5 million in 2025 to 35.6 million in 2050, driven by the ageing of the global population, and this increase in the CVD burden will largely be attributed to ASCVD [4]. According to the 2023 *Atlas of Cardiovascular Disease Statistics* from the European Society of Cardiology [3], healthcare and social care for CVDs represent a significant economic burden for the European Union (EU), reaching EUR 155 billion in 2021 in direct costs (11% of total EU healthcare expenditure), in addition to indirect costs (EUR 79 billion) resulting from lost working time for both patients and their caregivers, plus EUR 62 billion in lost productivity and early retirement of the affected population.

The main ASCVDs involve coronary (ischaemic) heart disease (CHD) [5,6], stroke [7], and peripheral arterial disease (PAD) [8]. CHD and stroke are the two leading causes of mortality, accounting for 14% and 7% in men, and 13% and 9% in women, respectively. CHD remains the leading cause of CVD, with the highest rates of age-standardised disability-adjusted life years [3,6]. Stroke remains the second leading cause of death worldwide, with an annual mortality rate of about 5.5 million [7]. PAD is the third most common form of ASCVD [3,8].

**Figure 1:**
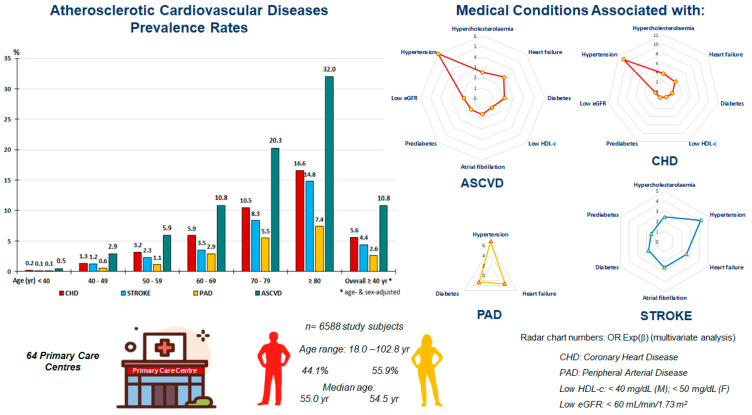
Graphical abstract: ASCVDs’ prevalence rates and their independent associations with cardiovascular–kidney–metabolic medical disorders.

Cardiovascular–kidney–metabolic (CKM) syndrome is defined as a complex health disorder due to the connections between heart disease, kidney disease, diabetes mellitus (DM), and obesity, leading to poor health outcomes [9,10]. It was developed to better understand the complexity of many interrelated CKM disorders compared with metabolic syndrome (MetS) [11] or cardiorenal syndrome [12], and to facilitate comprehensive assessments of the risks of ASCVD and chronic kidney disease (CKD). However, it is very complex to separately assess the severity of the multiple disorders included in CKM syndrome for ASCVD and CKD. On the other hand, we did not find recent population-based prevalence studies for ASCVDs in Spanish adults aged 18 to over 100 years. Therefore, the aims of this paper were to update the prevalence rates of ASCVDs among Spanish adults and evaluate their main relationships with the disorders included in CKM syndrome (Figure 1).

## 2. Materials and Methods

### 2.1. Study Design

**Figure 2:**
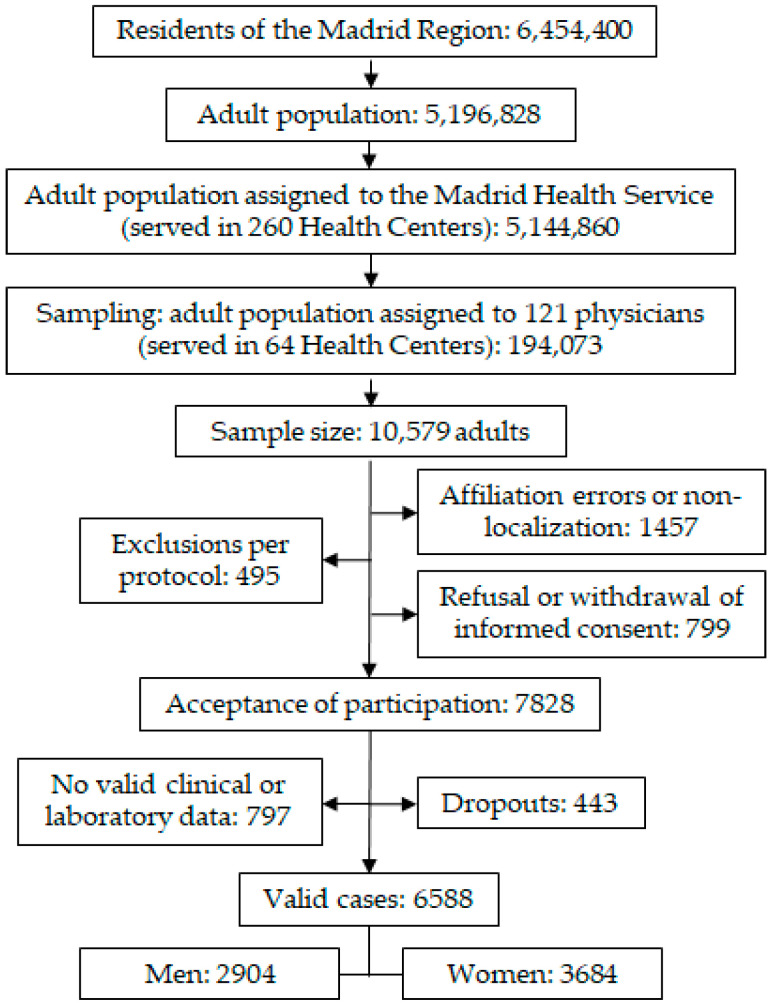
Flowchart for sampling and selection of study subjects.

The observational, cross-sectional, multicentre SIMETAP study was authorised by the Madrid Health Service (SERMAS, according to its acronym in Spanish), whose design was previously published in [13]. The flowchart for the sampling and enrolling of study subjects is shown in Figure 2. Healthcare for 99% of the adult population (5,144,860 people) from the Madrid Region (Spain) was provided in 260 SERMAS healthcare centres. This population was identified using the SERMAS database, which contains the numerical digits of each individual’s Health Identity Card (HIC). A simple random sample was performed using the “randbetween” function of the Microsoft Excel application among all people aged 18 or older assigned to 121 research physicians from 64 SERMAS health centres and identified by their respective HICs (194,073 adults). The sample size for this finite population was calculated considering *p* = 0.5 for the expected proportion, 25% for non-response, and 14% for losses and dropouts, with a confidence level of 95% (α error), and 2.4% for the confidence interval. The physicians conducted interviews with the participants and collected data from their electronic medical records in a real-world primary care setting.

In strict compliance with the protocol approved by the Research Ethics Committee (Code 05/2010RS), the inclusion criteria for the study subjects were as follows: adults aged 18 years or older, who had signed an informed consent form, and whose electronic medical records contained the clinical and laboratory data necessary to evaluate this study’s aims. Patients with schizophrenia, cognitive impairment, moderate or severe psychosis, dementia, or terminal illnesses, along with pregnant women, residents in nursing homes, and people who were participating in other clinical studies, were excluded. No generative artificial intelligence (GenAI) was used for the study design, data collection, interpretation, editing data, graphics, analysis, or text.

### 2.2. Assessment Variables

The primary outcomes assessed were the following diseases, recorded with their respective *International Classification of Diseases tenth revision, Clinical Modification* (ICD-10-CM) and/or *International Classification of Primary Care, second edition* (ICPC-2) codes [14,15], in the electronic medical records of the study subjects: CHD, including ischaemic heart disease, myocardial infarction (MI), coronary artery disease, acute coronary syndrome, acute and non-acute myocardial ischaemic syndromes, and coronary revascularisation (I20-I25; K74, K75, K76) [5,6]; stroke, including cerebral ischaemia, ischaemic stroke, transient ischaemic attack, subarachnoid haemorrhage, intracerebral haemorrhage, and intracranial haemorrhage (I60-I66, I66, I67; K89, K90K K91) [7]; PAD, including lower-extremity PAD, intermittent claudication (fatigue, cramping, aching, pain, or other discomfort of vascular origin in the muscles of the lower extremities that is consistently induced by walking and consistently relieved by rest, usually within approximately 10 min), or an ankle–brachial index ≤ 0.9 (I70.2, I73.9; K92) [8]; and ASCVD, including CHD, stroke, or PAD. The definitions and criteria of other clinical conditions, disorders, or diseases assessed in this paper are reported in detail in Table S1 (Supplementary Materials).

### 2.3. Statistical Analysis

Crude prevalence rates were determined for the overall adult population and for age groups. The direct method was used to calculate age- and sex-adjusted prevalence rates, initially determining the age-specific prevalence rates in the study population and the age distribution of the Spanish adult population according to the National Institute of Statistics census data, and then applying the age-specific rates of the study population to the age distribution of the Spanish adult population and summing the results. The frequency and percentage were determined for qualitative variables, and the mean and standard deviation (SD) for quantitative variables. Percentages and odds ratios (ORs) were reported with 95% confidence intervals (CIs). The chi-squared test or Fisher’s exact test was used to compare the results of the qualitative variables. To interpret the risk estimate of an event in the evaluated group versus the control group, the following effect sizes were considered: mild or minimum recommended (OR 2.0), moderate (OR 3.0), and strong (OR ≥ 4.0) [16]. The Shapiro–Wilk test was used to check whether the distribution of the results of the continuous variables fit to normal curves, and then we compared them using Student’s *t*-test or analysis of variance. Cohen’s *d* was used to assess the effect size of standardised mean differences, according to their proximity to the following absolute *d*-values: very small effect (≤0.1), small effect (0.1 to 0.2), moderate effect (0.3 to 0.7), large effect (0.8 to 1.1), and very large effect (≥1.2) [17].

The backward stepwise multivariate model was performed to evaluate the individual effects of clinical conditions on the dependent variables (CHD, stroke, PAD, and ASCVD). The variables that showed an association in the bivariate analyses up to a *p*-value < 0.10 were included in the multivariate model, except for erectile dysfunction (because it only affects men) and for MetS [11] (to avoid potential overadjustment and bias due to collinearity, because it includes five defining criteria that were evaluated separately). The *p*-value < 0.05 was used to determine the two-tailed statistical significance. The statistical analyses were performed with SPSS Statistics (version 25, IBM Corporation, Armonk, NY, USA).

## 3. Results

### 3.1. Prevalence Rates

A total of 6588 people (55.9% women) between 18.0 and 102.8 years of age (mean [SD] age: 55.1 [17.5] years) were assessed, with a non-significant age difference between men (55.3 [16.9] years) and women (55.0 [18.0] years) (*p* = 0.634).

The crude and adjusted prevalence rates of CHD, stroke, PAD, and ASCVD in both the 18-and-older and 40-and-older populations are shown in Table 1. All of these rates were significantly higher in men than in women. The age-group distributions of prevalence rates for CHD, stroke, PAD, and ASCVD increased precisely with age (*R^2^* > 0.98), according to polynomial functions (Figures S1–S4 [Supplementary Materials]). The age-specific prevalence rates for CHD and ASCVD were higher in men than in women for all age groups from 40 years of age (Figures S1 and S4 [Supplementary Materials]), between 50 and 69 years for stroke (Figure S2 [Supplementary Materials]), and from 60 years for PAD (Figure S3 [Supplementary Materials]).

### 3.2. Clinical Characteristics for Populations with and Without ASCVDs

The means (SD) of the clinical characteristics of the populations with CHD, stroke, PAD, and ASCVD are shown in Table 2 and Table S2 (Supplementary Materials). Differences in means and effect sizes between quantitative variables of populations with and without CHD, stroke, PAD, or ASCVD are shown in Tables S3–S6 (Supplementary Materials).

The differences in percentages between male and female populations with CHD, stroke, PAD, and ASCVD were 23.4% (95% CI 18.1–28.7), 7.4% (95% CI 1.1–13.7), 19.0% (95% CI 11.2–26.9), and 18.1% (95% CI 14.0–22.2), respectively. The differences in mean age between female and male populations with CHD, stroke, PAD, and ASCVD were 4.6 yr (*p* = 0.003), 4.3 yr (*p* = 0.011), 2.7 yr (*p* = 0.181), and 4.7 yr (*p* < 0.001), respectively. The differences in mean age between populations with and without CHD, stroke, PAD, and ASCVD were 17.8 yr, 18.9 yr, 17.9 yr, and 18.7 yr (*p* < 0.001), respectively.

The values for all assessed disorders and medical conditions were higher in populations with CHD, stroke, PAD, or ASCVD than in the respective populations without CHD, stroke, PAD, or ASCVD, except for total cholesterol (TC), low-density lipoprotein cholesterol (LDL-c), high-density lipoprotein cholesterol (HDL-c), and estimated glomerular filtration rate (eGFR) according to CKD-EPI [18], which were higher in the populations without ASCVDs (Tables S3–S6, Supplementary Materials).

Among the ASCVD population, 75.0% were on lipid-lowering treatment (LLT), 8.4% had LDL-c < 55 mg/dL, 13.1% had LDL-c between 55 and 69 mg/dL, 14.1% had non-HDL-c < 85 mg/dL, and 15.8% had non-HDL-c between 85 and 99 mg/dL.

### 3.3. CKM Disorders Related to ASCVDs

**Figure 3:**
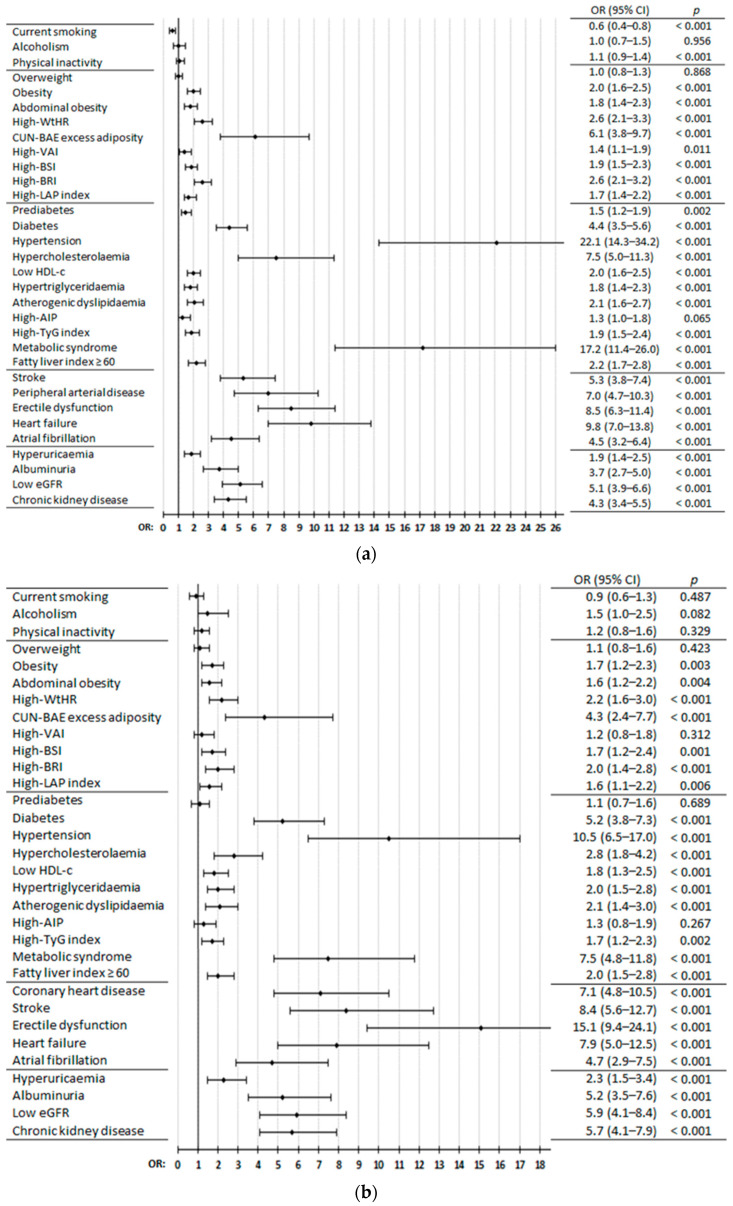

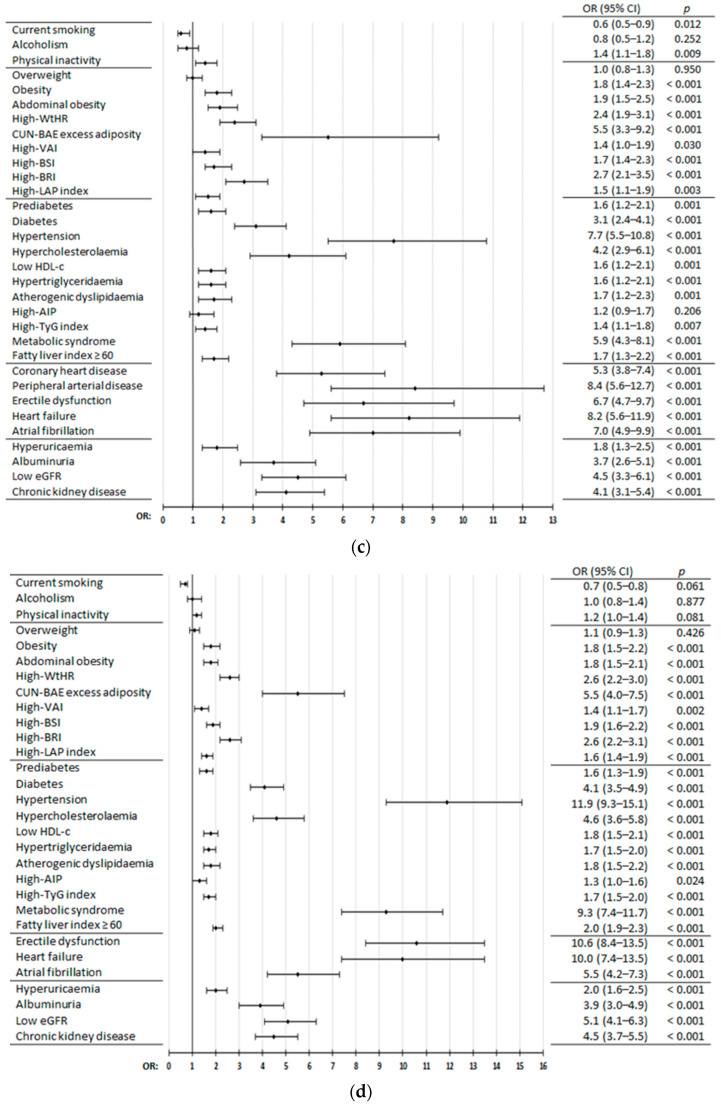
Diseases and medical conditions in populations with vs. without CHD (**a**), stroke (**b**), PAD (**c**), and ASCVD (**d**). CI: confidence interval; OR: odds ratio; *p*: *p*-value of the difference in percentage; AIP: atherogenic index of plasma; ASCVD: atherosclerotic cardiovascular disease; BRI: body roundness index; BSI: body shape index; CHD: coronary heart disease; CKD: chronic kidney disease; CUN-BAE: according to its acronym in Spanish, *Clínica Universitaria de Navarra*—Body Adiposity Estimator; eGFR: estimated glomerular filtration rate; HDL-c: high-density lipoprotein cholesterol; LAP: lipid accumulation product index; PAD: peripheral arterial disease; TyG: triglyceride–glucose index; VAI: visceral adiposity index; WtHR: waist-to-height ratio. The definitions of diseases or medical conditions are shown in Table S1 (Supplementary Materials).

The clinical conditions related to excess or dysfunctional adiposity that showed association with ASCVDs were obesity [19], abdominal obesity [11], and high values of the waist-to-height ratio (WtHR) [20], CUN-BAE (according to its acronym in Spanish, *Clínica Universitaria de Navarra*—Body Adiposity Estimator) [21,22], visceral adiposity index (VAI) [23,24], body shape index (BSI) [22,25], body roundness index (BRI) [22,26], and lipid accumulation product (LAP) index [24,27], highlighting CUN-BAE excess adiposity (OR: 4.3 to 6.1) (Figure 3a–d, Tables S6–S9 [Supplementary Materials]). DM [28], HTN [29], hypercholesterolaemia [30], low HDL-c [30], HTG [30], atherogenic dyslipidaemia, high triglyceride–glucose (TyG) index [24,31], MetS [11], fatty liver index (FLI) ≥ 60 [32], and hyperuricaemia [33] showed associations with ASCVDs, highlighting DM (OR: 3.1 to 5.2), hypercholesterolaemia (OR: 2.8 to 7.5), and especially MetS (OR: 5.9 to 17.1) and HTN (OR: 7.7 to 22.1). Prediabetes [28] was associated with CHD, stroke, and ASCVD, but not with PAD. High atherogenic index of plasma (AIP) [34] was associated only with ASCVD (Figure 3a–d, Tables S7–S10 [Supplementary Materials]). Albuminuria (urine albumin–creatinine ratio ≥ 30 mg/g), low eGFR (< 60 mL/min/1.72 m^2^), and CKD [35] were strongly associated with CHD, stroke, PAD, and ASCVD, highlighting low eGFR (OR: 4.5 to 5.9) (Figure 3a–d, Tables S6–S9 [Supplementary Materials]).

Having one ASCVD was strongly associated with the other two ASCVDs: CHD (OR: 5.3 for stroke; 7.0 for PAD), stroke (OR: 5.3 for CHD; 8.4 for PAD), and PAD (OR: 7.1 for CHD; 8.4 for stroke). Other CVDs, such as erectile dysfunction (OR: 6.7 to 15.1)—considered an early manifestation of ASCVD [36]—HF [37] (OR: 7.9 to 10.0), and AF [38,39] (OR: 4.5 to 7.0), were also strongly associated with ASCVDs, highlighting HF (Figure 3a–d, Tables S7–S10 [Supplementary Materials]).

### 3.4. Independent Associations of CKM Disorders with ASCVDs

**Figure 4:**
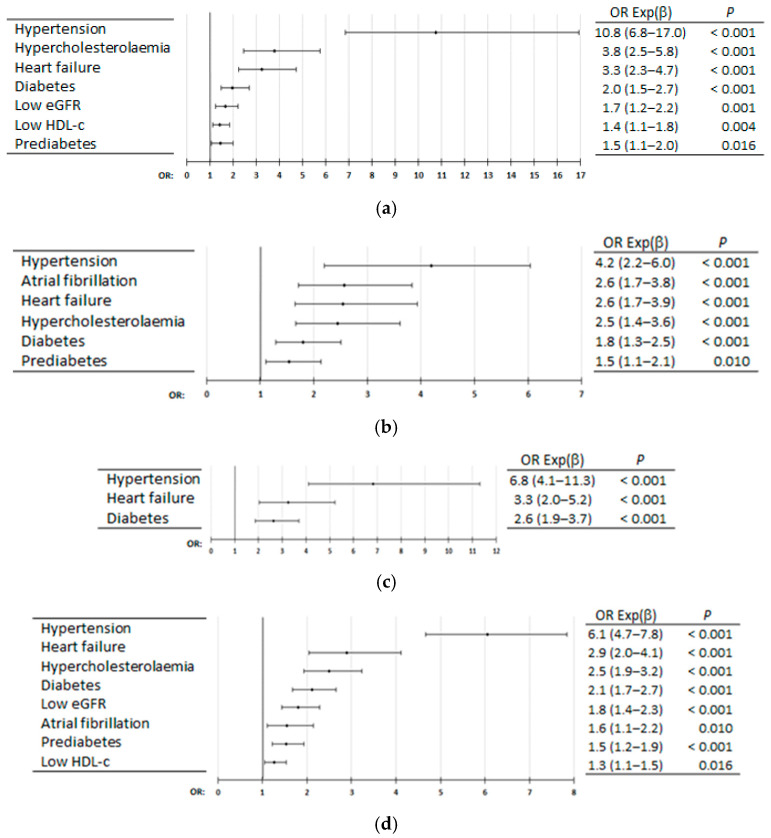
Multivariate analysis of diseases and medical conditions for coronary heart disease (**a**), stroke (**b**), peripheral arterial disease (**c**), and atherosclerotic cardiovascular disease (**d**). OR Exp (β): odds ratio (95% confidence interval); *p*: *p*-value of Wald test with one degree of freedom; eGFR: estimated glomerular filtration rate; HDL-c: high-density lipoprotein cholesterol.

Multivariate analyses showed that HTN, DM, and HF were independently associated with CHD, stroke, PAD, and ASCVD, highlighting HTN. In addition, hypercholesterolaemia and prediabetes were also independently associated with CHD, stroke, and ASCVD; low HDL-c and low eGFR were independently associated with CHD and ASCVD; and AF was independently associated with stroke and ASCVD (Figure 4a–d, Table S11a–d [Supplementary Materials]).

## 4. Discussion

### 4.1. ASCVDs’ Prevalence Rates

Studies show different estimates of ASCVDs’ prevalence rates because they differ in the types of sampling in populations with different age ranges. According to NHANES data from 2017 to 2020, among United States (US) adults ≥ 20 years of age, the prevalence of CHD and stroke was 7.1% and 3.3%, respectively [40]. PAD’s prevalence has continued to increase in recent years, likely due to the ageing population. The 2019 Global Burden of Disease Study showed that the global prevalence of PAD in people aged 40 years and older was 1.5% (1.0% in men and 2.0% in women). PAD is uncommon in those younger than 40 years of age, affecting one in ten people aged 70 years and one in six people aged 80 years and older [41]. The prevalence of PAD in the US among individuals ≥ 40 years of age reached 10.7% between 2003 and 2008, according to a report from the American Heart Association (AHA) [42]. Age is associated with CKM disorders, typically including DM, ASCVD, and CKD [43]. Our data showed that ASCVD events were rare in people younger than 40 years, and ASCVD’s prevalence was very similar to the AHA report [42] in people aged 40 years and older. The ASCVD prevalence rates increased precisely with age according to simple second-degree polynomial functions, and they were higher in men than in women from age 40 years for CHD and ASCVD, from age 50 to 69 years for stroke, and from age 60 years for PAD. Some authors report that cardiovascular events in women usually occur about five to ten years later than in men [44,45]. On the other hand, our results also showed a higher mean age among women with ASCVD than among men, although the difference did not exceed five years (74.9 and 70.2 years, respectively).

### 4.2. Unhealthy Lifestyles

Unhealthy lifestyles promote the development of ASCVD [46,47,48]. The extensive knowledge about ASCVD recommends focusing on the so-called primordial prevention [49], which consists of the comprehensive prevention of all CVD risk factors, since their absence reduces the incidence of cardiovascular events and improves life expectancy [50]. In this regard, our results were contradictory, since both physical inactivity and high alcohol consumption were not associated with ASCVD, and smoking rates were lower in the population with CHD or stroke, probably due to the greater emphasis of healthcare providers on improving healthy lifestyle behaviours and encouraging smoking cessation in patients with ASCVD.

### 4.3. Factors Related to Adiposity

CKM syndrome is based on excess/dysfunctional adiposity [9,10]. Although obesity is strongly associated with an increased risk of developing ASCVD, this excess risk is partly mediated and increased by other major obesity-related risk factors, such as DM, HTN, or dyslipidaemia [51]. Our study data showed that being overweight was not associated with ASCVDs, and that almost all other adiposity-related clinical parameters showed a weak association with CHD, stroke, PAD, and ASCVD. Furthermore, none of these parameters showed an independent association with any of the ASCVDs in the multivariate analyses performed separately for each of them. Only CUN-BAE excess adiposity [22] showed strong associations with all ASCVDs. These results could suggest that other parameters, such as simple obesity defined by body mass index [19], abdominal obesity [11], high VAI [22], high BSI [22], or high LAP index [24], could be relegated to a secondary level to consider excess/dysfunctional adiposity, compared to high WtHR [20] or high BRI [22], but especially compared to CUN-BAE excess adiposity [22].

### 4.4. Cardiometabolic Risk Disorders

Patients with type 2 DM often have multiple risk factors for ASCVD. DM and prediabetes are independent risk factors for ASCVD, doubling the risk of ASCVD [52]. Our results showed that DM was independently associated with all ASCVDs, and prediabetes was associated with CHD, stroke, and ASCVD, but not with PAD. In line with already known data [53], our study confirms that MetS [11] was strongly associated with all ASCVDs, since all of the criteria that define it were also associated with all of them.

HTN remains the leading CVD risk factor worldwide, contributing to 10.8 million deaths in 2019 [2]. The Prospective Studies Collaboration Group found that the risk of fatal CHD or stroke doubled for each 20 mmHg elevation in SBP [54]. The results of both our bivariate and multivariate analyses confirmed that HTN was the risk factor most strongly associated with all of the ASCVDs.

Hypercholesterolaemia, defined as increased levels of both LDL-c and non-HDL-c (containing apo-B lipoproteins), is the primary cause of ASCVD. This has been demonstrated beyond any doubt by Mendelian randomisation, epidemiological, and intervention studies [55]. On the other hand, some studies have shown associations between HTG and an increased ASCVD risk [56,57], although there are also other lipid and metabolic disorders that could bias this relationship [58]. A high TyG index may be independently associated with CHD and stroke [59]. Our results showed that although hypercholesterolaemia maintained a strong association with all ASCVDs, altered lipid profile parameters had mild or no associations with ASCVDs, probably because 75% of ASCVD patients were on LLT. Multivariate analyses confirmed that hypercholesterolaemia was independently associated with CHD, stroke, and ASCVD, highlighting CHD, and that low HDL-c [30] was independently associated with CHD and ASCVD.

On the other hand, CKM syndrome [9,10] does not include metabolically associated steatotic liver disease (MASLD), a medical condition closely linked to insulin resistance and obesity that contributes to the progression of atherosclerosis and the worsening of HF and CKD. Including MASLD would justify expanding the current CKM syndrome framework to another model called cardiovascular–renal–hepatic–metabolic [60]. However, our results showed that FLI ≥ 60, a parameter that can be used to rule in steatotic liver disease (specificity 86%; positive likelihood ratio: 4.3) [32], was only mildly associated with ASCVD. CKM syndrome [9,10] also does not include hyperuricaemia, even though it stimulates the production of pro-inflammatory molecules that promote the pathogenesis of atherosclerosis [61,62]. Our results showed mild associations between hyperuricaemia and CHD, stroke, PAD, and ASCVD.

### 4.5. Other Cardiovascular Diseases

CVDs represent the leading cause of death worldwide [1,2,3]. CHD is the most common cause of HF, so it should always be considered in patients presenting onset HF [37]. AF and HF frequently coexist, and either can predispose to the development of the other [38,39]. There is a bidirectional association between AF and stroke. The Rotterdam study showed that subclinical carotid atherosclerosis was associated with AF incidence [63]. The Framingham study showed that the presence of AF was a factor associated with the incidence of stroke [64].

Our multivariate analyses showed that HF was independently associated with CHD, stroke, PAD and ASCVD, and that AF was independently associated with stroke.

### 4.6. Chronic Kidney Disease

ASCVD is the main cause of mortality in patients with CKD [65], and CKD is an amplifier of ASCVD risk, since both albuminuria and low eGFR independently increase the risk of ASCVD and cardiovascular death [35]. Our results confirmed that low eGFR, albuminuria, and CKD were strongly associated with all ASCVDs, and that low eGFR showed an independent association with both CHD and ASCVD in multivariate analysis. It should be noted that moderate, high, and very high risk of CKD (understood as the risk of acute kidney injury, kidney failure replacement therapy, all-cause mortality, and cardiovascular events [35]) were strongly associated with all ASCVDs, highlighting high-risk and very high-risk CKD. Based on these results, we believe that not only very high-risk CKD patients but also high-risk CKD patients should be included in advanced stage 3 CKM syndrome.

### 4.7. Other Clinical Considerations

The SANTORINI Spain study [66] showed that 27.9% of patients enrolled in 82 Spanish health centres (90% hospitals), assigned as very high risk according to the clinical judgment of their physicians (67.4% had ASCVD) and followed-up for 12 months, achieved a LDL-c < 55 mg/dL. The EUROASPIRE V [67] survey showed that only 29% of CHD patients achieved an LDL-c < 70 mg/dL six months after hospitalisation for a coronary event. Our results were very similar among the CHD population. It should be noted that the therapeutic targets for LDL-c and non-HDL-c in the EUROSPIRE V survey and our study were less stringent than the current ones, because the participants were enrolled prior to the 2019 ESC/EAS Guidelines for the management of dyslipidaemias [68].

Patients with CKM syndrome may have a three-fold-increased risk of all-cause mortality compared to those with stage 0, and up to a ten-fold-increased risk of cardiovascular mortality in those with stage 4 [69]. Understanding the interactions of the multiple disorders of CKM syndrome is highly complex. The CKM syndrome staging aims to understand their evolutionary interrelationship, which may begin with excess or dysfunctional adiposity in stage 1 and/or with other CKM disorders in stage 2 [9,10]. Furthermore, the complex aetiology of the multiple disorders of CKM syndrome may favour the development or occurrence of other CKM disorders before or after the first cardiovascular event. Early detection of CKM disorders is critical for the prevention of ASCVD. These findings emphasise primordial prevention efforts, such as the Life’s Essential 8 of the AHA, which focuses on control of the following major health factors: diet, physical activity, smoking, sleep health, obesity, blood lipids, blood glucose, and blood pressure [70].

### 4.8. Limitations and Strengths

The limitations of our study included the inability to estimate incidence rates or to preclude definitive conclusions about causal relationships due to its cross-sectional observational design, heterogeneity in measurement accuracy due to different laboratory equipment and inter-interviewer variability, and underestimation of prevalence rates because the excluded population per protocol was not considered. There were few values that were not reported in some variables, occurred at random, and were proportionally similar in the comparison groups, although this could imply minimal confounding in the comparative analysis between subjects with and without ASCVDs. Associations between various factors or clinical conditions and the presence of ASCVD should be interpreted with scientific caution, as comparisons could increase the risk of obtaining a statistically significant association by chance (alpha error), so their respective effect sizes must be carefully assessed. Comments could be made regarding CKD risk [35] stratification, but no comment could be made regarding cardiovascular risk, because all subjects with ASCVDs were at very high risk according to the 2021 ESC Guidelines on cardiovascular disease prevention in clinical practice [48]. The therapeutic effect of the greater intensity of lipid-lowering, antihypertensive, and hypoglycaemic therapies in patients with ASCVD should also be considered, as this influences the reduction in the outcome values and may be especially relevant for lipid profile parameters.

Among the strengths of this study is the inclusion of a large number of people between 18 and 102 years of age. Sampling was adopted using a random population-based approach and not random sampling among patients who regularly attended the clinic, so as to avoid selection bias and be consistent with a true approximation of population prevalence. This paper updates the prevalence rates of ASCVDs in the adult population. We are convinced that assessing the epidemiological magnitude of ASCVDs is essential for better planning health prevention policies and optimising the available resources. We believe that this paper not only provides comprehensive analyses of the multiple disorders included in the four stages of CKM syndrome but also succeeds in highlighting those most relevant to each of the ASCVDs, both evaluated separately and as a whole. The data reported herein are biologically plausible and consistent with the available scientific information, and they could contribute to a better understanding of the clinical characteristics of ASCVDs and their associations with many CKM disorders.

## 5. Conclusions

The prevalence of ASCVDs in the Spanish population remains high, probably due to poor control of the factors associated with them. More than one in ten people aged years and older suffer from CHD, stroke, or PAD. ASCVD is more common in men than in women approximately five years older. CHD, stroke, or PAD is strongly associated with the presence of any of the other ASCVDs. Eight CKM components are independently associated with ASCVD. HTN, HF, and DM are the main common factors associated with CHD, stroke, and PAD, highlighting HTN and HF for all of them, in addition to hypercholesterolaemia for CHD and stroke, and specifically, AF for stroke. High-risk CKD patients should also be included in advanced stage 3 CKM syndrome. Early detection and close management of all of these CKM disorders are essential to comprehensively reduce the development of ASCVDs and the incidence of new atherosclerotic cardiovascular events.

## Figures and Tables

**Table 1:** Prevalence rates for CHD, stroke, PAD, and ASCVD.

	Crude Prevalence Rates				Age-Adjusted Prevalence Rates		
	Male * % (95% CI)	Female * % (95% CI)	*p*	Overall * % (95% CI)	Male (%)	Female (%)	Overall (%)
CHD (≥18 yr)	7.3 (6.4–8.3)	2.9 (2.4–3.5)	<0.001	4.9 (4.4–5.4)	5.3	2.4	3.8
CHD (≥40 yr)	9.1 (8.0–10.3)	3.8 (3.1–4.5)	<0.001	6.2 (5.5–6.9)	8.1	3.5	5.6
Stroke (≥18 yr)	4.4 (3.7–5.2)	3.3 (2.7–3.9)	0.021	3.8 (3.3–4.3)	3.2	2.8	3.0
Stroke (≥40 yr)	5.6 (4.6–6.5)	4.2 (3.5–5.0)	0.031	4.8 (4.2–5.4)	4.9	4.0	4.4
PAD (≥18 yr)	3.2 (2.6–3.9)	1.5 (1.1–1.9)	<0.001	2.3 (1.9–2.6)	2.3	1.3	1.8
PAD (≥40 yr)	4.1 (3.3–4.9)	1.9 (1.4–2.4)	<0.001	2.9 (2.4–3.3)	3.5	1.8	2.6
ASCVD (≥18 yr)	12.8 (11.6–14.0)	6.6 (5.8–7.4)	<0.001	9.3 (8.6–10.0)	9.3	5.5	7.3
ASCVD (≥40 yr)	16.0 (14.5–17.5)	8.4 (7.4–9.4)	<0.001	11.8 (11.0–12.7)	14.2	7.9	10.8

* No. population ≥ 18 yr: 2904 men, 3684 women, 6588 overall; No. population ≥ 40 yr: 2308 men, 2827 female, 5135 overall; CI: confidence interval; *p*: *p*-value of difference in percentages; ASCVD: atherosclerotic cardiovascular disease; CHD: coronary heart disease; PAD: peripheral arterial disease; yr: years old.

**Table 2:** Clinical characteristics for populations with CHD, stroke, PAD, and ASCVD.

	CHD *	Stroke *	PAD *	ASCVD *
	Mean (SD)	Mean (SD)	Mean (SD)	Mean (SD)
Age yr	72.1 (12.8)	73.3 (13.4)	72.6 (12.1)	72.1 (13.1)
BMI kg/m^2^	29.6 (5.4)	29.0 (5.1)	28.7 (4.7)	29.2 (5.2)
WC cm	100.5 (13.4)	97.8 (12.9)	98.2 (12.4)	99.0 (13.2)
CUN-BAE adiposity	36.6 (8.0)	37.8 (8.1)	36.1 (7.7)	36.8 (8.0)
SBP mm Hg	127.0 (15.5)	128.1 (15.3)	128.3 (15.2)	127.5 (15.3)
DBP mm Hg	73.3 (9.8)	74.1 (9.7)	73.0 (9.0)	73.7 (9.6)
FPG mg/dL	110.3 (32.6)	104.7 (27.6)	107.6 (33.3)	108.0 (32.0)
HbA1c %	6.20 (1.04)	5.98 (0.96)	6.18 (0.98)	6.11 (1.02)
TC mg/dL	163.8 (37.6)	180.6 (41.1)	170.1 (39.3)	173.0 (40.1)
HDL-c mg/dL	50.0 (14.9)	52.1 (13.7)	50.5 (13.5)	51.0 (14.1)
LDL-c mg/dL	88.2 (31.0)	103.8 (35.4)	93.2 (31.9)	96.5 (34.0)
TG mg/dL	128.8 (70.4)	125.5 (71.5)	131.3 (69.2)	128.6 (72.7)
GGT U/L	44.2 (53.5)	39.8 (48.0)	43.7 (43.6)	41.6 (48.5)
FLI 0–100	60.5 (27.1)	55.3 (27.0)	57.8 (26.5)	57.7 (27.3)
Creatinine mg/dL	1.02 (0.48)	0.93 (0.30)	1.07 (0.65)	0.98 (0.44)
eGFR mL/min/1.73 m^2^	72.5 (20.6)	74.4 (21.0)	70.0 (21.5)	73.7 (20.7)
uACR mg/g	39.4 (139.7)	37.2 (119.6)	53.0 (124.0)	40.1 (129.5)

ASCVD: atherosclerotic cardiovascular disease; BMI: body mass index; CHD: coronary heart disease; CUN-BAE: according to its acronym in Spanish, *Clínica Universitaria de Navarra*—Body Adiposity Estimator; DBP: diastolic blood pressure; eGFR: estimated glomerular filtration rate; FPG: fasting plasma glucose; FLI: fatty liver index; GGT: gamma-glutamyl transferase; HbA1c: glycated haemoglobin A1c; HDL-c: high-density lipoprotein cholesterol; LDL-c: low-density lipoprotein cholesterol; PAD: peripheral arterial disease; SBP: systolic blood pressure; SD: standard deviation; TC: total cholesterol; TG: triglyceride; uACR: urine albumin–creatinine ratio; WC: waist circumference. * No. CHD: 321; No. stroke: 250; No. PAD: 150; No. ASCVD: 615.

## Data Availability

The original contributions presented in this study are included in the article/Supplementary Materials.

## Supplementary Materials

The following supporting information can be downloaded at https://www.mdpi.com/article/10.3390/jcm14113940/s1: Table S1: Definitions of diseases and clinical conditions criteria; Figure S1: Age-specific prevalence rates of coronary heart disease; Figure S2: Age-specific prevalence rates of stroke; Figure S3: Age-specific prevalence rates of peripheral arterial disease; Figure S4: Age-specific prevalence rates of atherosclerotic cardiovascular disease; Table S2: Clinical characteristics of populations with ASCVDs; Table S3: Quantitative clinical variables in populations with and without CHD; Table S4: Quantitative clinical variables in populations with and without stroke; Table S5: Quantitative clinical variables in populations with and without PAD; Table S6: Quantitative clinical variables in populations with and without ASCVD; Table S7: Diseases and medical conditions in populations with and without CHD; Table S8: Diseases and medical conditions in populations with and without stroke; Table S9: Diseases and medical conditions in populations with and without PAD; Table S10: Diseases and medical conditions in populations with and without ASCVD; Table S11: Multivariate analysis of diseases and medical conditions for CHD (a), stroke (b), PAD (c), and ASCVD (d).

## Abbreviations

The following abbreviations are used in this manuscript:

AF	Atrial fibrillation
AHA	American Heart Association
AIP	Atherogenic index of plasma
ALT	Alanine aminotransferase
ASCVD	Atherosclerotic cardiovascular disease
AST	Aspartate aminotransferase
BRI	Body roundness index
BSI	Body shape index
CHD	Coronary (ischaemic) heart disease
CI	Confidence interval
CKD	Chronic kidney disease
CKM	Cardiovascular–kidney–metabolic
CUN-BAE	*Clínica Universitaria de Navarra*—Body Adiposity Estimator
CVD	Cardiovascular disease
DBP	Diastolic blood pressure
DM	Diabetes mellitus
eGFR	Estimated glomerular filtration rate
FLI	Fatty liver index
FPG	Fasting plasma glucose
HbA1c	Glycated haemoglobin A1c
HDL-c	High-density lipoprotein cholesterol
HTG	Hypertriglyceridaemia
HTN	Arterial hypertension
LAP	Lipid accumulation product index
LDL-c	Low-density lipoprotein cholesterol
LLT	Lipid-lowering drug therapy
MASLD	Metabolically associated steatotic liver disease
MetS	Metabolic syndrome
MI	Myocardial infarction
OR	Odds ratio
PAD	Peripheral arterial disease
SBP	Systolic blood pressure
SD	Standard deviation
TC	Total cholesterol
TG	Triglyceride
TyG	Triglyceride–glucose index
VAI	Visceral adiposity index
WtHR	Waist-to-height ratio
