# Supplementary material for: From the Cardiovascular–Kidney–Metabolic Disorders to the Atherosclerotic Cardiovascular Diseases: Their Prevalence Rates and Independent Associations in the SIMETAP Study
Source: J Clin Med. 2025 Jun 3;14(11):3940. doi: 10.3390/jcm14113940 (PMC12155652; doi:10.3390/jcm14113940)
Supplement: Supplementary file 1 [file jcm-14-03940-s001.zip › jcm-3655155-supplementary.pdf]

From the Cardiovascular-Kidney-Metabolic Disorders to the Atherosclerotic Cardiovascular Diseases: Their Prevalence Rates and Independent Associations in the SIMETAP Study

SUPPLEMENTARY MATERIALS

Table S1. Definitions of diseases and clinical conditions criteria

Figure S1. Age-Specific Prevalence Rates of Coronary Heart Disease

Figure S2. Age-Specific Prevalence Rates of Stroke

Figure S3. Age-Specific Prevalence Rates of Peripheral Arterial Disease

Figure S4. Age-Specific Prevalence Rates of Atherosclerotic Cardiovascular Disease

Table S2. Clinical characteristics for populations with ASCVDs

Table S3. Quantitative clinical variables in populations with and without CHD

Table S4. Quantitative clinical variables in populations with and without Stroke

Table S5. Quantitative clinical variables in populations with and without PAD

Table S6. Quantitative clinical variables in populations with and without ASCVD

Table S7: Diseases and medical conditions in populations with and without CHD

Table S8: Diseases and medical conditions in populations with and without Stroke

Table S9: Diseases and medical conditions in populations with and without PAD

Table S10: Diseases and medical conditions in populations with and without ASCVD

Table S11: Multivariate analysis of diseases and medical conditions for CHD (a), for Stroke (b), for PAD (c), and for ASCVD (d).

Table S1. Definitions of diseases and clinical conditions criteria

**Morbidities, diseases or clinical conditions**	**Definitions and criteria**
Current smoking	Any amount of tobacco use in the previous year.
Alcoholism (excessive alcohol consumption)	> 21 standard drink units (SDU) of alcohol consumption per week (male), > 14 SDU per week (female).<br>1 SDU is equivalent to 8 g or 10 mL of ethanol.
Physical inactivity [46]	Lack of moderate-intensity physical activity (e.g., brisk walking) < 150 minutes a week, or vigorous-intensity physical activity (e.g., jogging) < 75 minutes a week (or less of equivalent combination of both), or muscle strengthening exercises < 2 days a week.
Body mass index (BMI) [19]	BMI was calculated as weight in kilograms divided by the square of height in meters.
Overweight [19]	BMI 25.0–29.9 kg/m^2 (International Classification of Diseases, 10th Revision, Clinical Modification [ICD-10-CM]: E66.3; International Classification of Primary Care, 2nd edition [ICPC-2]: T83)*
Obesity [19]	BMI ≥ 30 kg/m^2 (ICD-10-CM: E66.9; ICPC-2: T82)*
Waist circumference (WC)	WC determined with the subject standing using a flexible tape measure adjusted without compressing the skin, after the participant exhaled a normal breath, locating the upper edge of the iliac crests and above that point surrounding the waist parallel to the floor.
Abdominal (or central) obesity (AO) [11]	WC ≥ 102 cm in men or ≥ 88 cm in women
Waist-to-height ratio (WtHR)	WtHR is calculated as WC measurement divided by height measurement, both in centimetres.
High-WtHR [20].	WtHR ≥ 0.60 for both men and women
CUN-BAE adiposity or CUN-BAE body fat index [21]	CUN-BAE (according to its acronym in Spanish, *Clínica Universitaria de Navarra* - Body Adiposity Estimator) body fat index:<br>• Men: − 44.988 + (0.503 x age) + (3.172 x BMI) − (0.026 x BMI^2) − (0.02 x BMI x age) + (0.00021 x BMI^2 x age)<br>• Women: − 44.988 + (0.503 x age) + 10.689 + (3.172 x BMI) − (0.026 x BMI^2) + (0.181 x BMI) − (0.02 x BMI x age) − (0.005 x BMI^2) + (0.00021 x BMI^2 x age)
CUN-BAE excess adiposity (EA) [21]	CUN-BAE values ≥ 30 in men and ≥ 35 in women were considered to define excessive increase in body fat or excess adiposity (EA)
Visceral adiposity index (VAI) [23]	VAI = {WC/[39.68 + (1.88 × BMI)]} × (TG/1.03) × (1.31/HDL-c) for men<br>VAI = {WC/[36.58 + (1.89 × BMI)]} × (TG/0.81) × (1.52/HDL-c) for women<br>WC is expressed in centimetres, BMI in kg/m^2, TG and HDL-c in mmol/L.

High-VAI [22]	High-VAI cut-off points for detecting MetS: ≥ 2.36 for men; ≥ 2.92 for women
Body shape index (BSI) [25]	BSI = WC / ($BMI^{2/3}$ x height $^{1/2}$)<br>Units are meters for WC and height, kg/m^2 for BMI, and $m^{11/6}$ $kg^{-2/3}$ for BSI
High-BSI [22]	High-BSI cut-off points for risk increase of adiposity: ≥ 0.084 for men and ≥ 0.082 for women
Body roundness index (BRI) [26]	BRI = 364.2 − 365.5 x {1 − $[(WC/2\pi)/(0.5 \text{ x height})]^2\}^{0:5}$<br>WC and height are expressed in centimetres.
High-BRI [22]	High-BRI cut-off points for risk increase of adiposity: ≥ 5.69 for men and ≥ 5.77 for women
Lipid accumulation product (LAP) [27]	LAP index is a calculation used to assess the risk of excessive lipid accumulation in the abdominal region.<br>LAP = (WC − 65) x TG for men<br>LAP = (WC − 58) x TG for women<br>WC is expressed in centimetres, and TG in mmol/L
High-LAP [24]	High-LAP cut-off points: ≥ 59.85 for men; ≥ 53.06 for women
Arterial hypertension (HTN) [29]	Systolic blood pressure (SBP) ≥ 140 mmHg and/or diastolic blood pressure (DBP) ≥ 90 mmHg, using the average of two or more readings obtained on two or more occasions, or being on blood pressure-lowering drug therapy (BPLT) (ICD-10-CM: I10, I15; ICPC-2: K86, K87)*
Pulse pressure	SBP – DBP (mmHg)
Diabetes mellitus (DM) [28]	It was defined according to the American Diabetes Association (ADA) criteria: fasting plasma glucose (FPG) ≥ 126 mg/dL (7.0 mmol/L) or glycated haemoglobin A_{1c} (HbA_{1c}) ≥ 6.5 % (in International Federation of Clinical Chemistry and Laboratory Medicine [IFCC] units) (≥ 48 mmol/mol) or plasma glucose ≥ 200 mg/dL (11.1 mmol/L) at any time or with oral glucose tolerance test (ICD-10-CM: E10, E11; ICPC-2: T89, T90)*<br>To convert from mg/dL to mmol/L, multiply by 0.05556<br>To convert from % (DCCT) to mmol/mol (IFCC), subtract 2.15 and multiply by 10.929
Prediabetes [28]	It was defined according to the ADA criteria: FPG between 100 and 125 mg/dL or HbA_{1c} between 5.7% and 6.4% (ICD-10-CM: R73.09; ICPC-2: A91)*<br>To convert from mg/dL to mmol/L, multiply by 0.05556<br>To convert from % (DCCT) to mmol/mol (IFCC), subtract 2.15 and multiply by 10.929
Estimated average glucose (eAG)	28.7 x HbA_{1c} – 46.7 (mg/dL)<br>To convert from mg/dL to mmol/L, multiply by 0.05556

Hypercholesterolaemia [30]	Fasting plasma total cholesterol (TC) levels ≥ 200 mg/dL (≥ 5.17 mmol/L) (ICD-10-CM: E78; ICPC-2: T93)* or being on lipid-lowering drug therapy (LLT) to achieve cholesterol targets.<br>To convert from mg/dL to mmol/L, multiply by 0.02586
Hypertriglyceridaemia (HTG) [30]	Fasting plasma triglycerides (TG) levels ≥ 150 mg/dL (≥ 1.69 mmol/L) (ICD-10-CM: E78; ICPC-2: T93)* or being on triglycerides lowering specific drug therapy.<br>To convert from mg/dL to mmol/L, multiply by 0.01129
Low high-density lipoprotein cholesterol (HDL-c) [30]	HDL-c < 40 mg/dL (< 1.03 mmol/L) (for men)<br>HDL-c < 50 mg/dL (< 1.29 mmol/L) (for women)<br>To convert from mg/dL to mmol/L, multiply by 0.02586
Atherogenic dyslipidaemia	HTG and low HDL-c
Non-high-density lipoprotein cholesterol (Non-HDL-c)	TC – HDL-c<br>To convert from mg/dL to mmol/L, multiply by 0.02586
Low-density lipoprotein cholesterol (LDL-c)	TC – HDL-c – (TG/5) mg/dL (not valid for patients with TG > 400 mg/dL)<br>TC – HDL-c – (TG/2.2) mmol/L (not valid for patients with TG > 4.51 mmol/L)<br>LDL-c, TC, HDL-c: To convert from mg/dL to mmol/L, multiply by 0.02586<br>TG: To convert from mg/dL to mmol/L, multiply by 0.01129
Residual cholesterol (RC)	Very low-density lipoproteins cholesterol (VLDL-c) and remnants.<br>RC= TC – HDL-c – LDL-c<br>LDL-c, TC, HDL-c: To convert from mg/dL to mmol/L, multiply by 0.02586
Atherogenic index of plasma (AIP) [34]	AIP = log (TG / HDL-c)<br>TG and HDL-c are expressed in mmol/L<br>AIP values between −0.3 to 0.1 are associated with a low CVD risk<br>AIP values between > 0.1 and 0.24 are associated with a medium CVD risk<br>AIP values > 0.24 are considered high risk for CVD
Triglyceride-glucose (TyG) index [31]	TyG index = Ln (TG x FPG/2)<br>TG and FPG are expressed in mg/dL
High-TyG [24]	High TyG cut-off points for detecting MetS: ≥ 8.77 for men and ≥ 8.70 for women

Metabolic syndrome (MetS) [11]	MetS according to 2009 Joint Statement of International Diabetes Federation Task Force on Epidemiology and Prevention, National Heart, Lung, and Blood Institute, American Heart Association, World Heart Federation, International Atherosclerosis Society, and International Association for the Study of Obesity.<br>MetS diagnosis is identify by the presence of three or more of the following criteria for the European population:<br>• Increased WC (≥ 102 cm for men; ≥ 88 cm for women)<br>• TG ≥ 150 mg/dL (≥ 1.7 mmol/L)<br>• HDL-c < 40 mg/dL (< 1.03 mmol/L) (males); < 50 mg/dL (< 1.29 mmol/L) (females)<br>• SBP ≥ 130 mmHg or DBP ≥ 85 mmHg (antihypertensive drug treatment in a patient with a history of HTN is an alternate indicator)<br>• FPG ≥ 100 mg/dL (≥ 5.6 mmol/L) (drug treatment for elevated glucose is an alternate indicator)
Fatty liver index (FLI) [32]	$FLI = (e^{0.953*\log_e(TG) + 0.139 \times BMI + 0.718 \times \log_e(GGT) + 0.053 \times \text{waist circumference} - 15.745}) / (1 + e^{0.953*\log_e(TG) + 0.139 \times BMI + 0.718 \times \log_e(GGT) + 0.053 \times \text{waist circumference} - 15.745}) \times 100$<br>$\log_e$ = natural logarithm; GGT = gamma-glutamyl-transferase<br>A value FLI between 0 and 30 can be used to rule out steatotic liver disease (SLD) (sensitivity: 87%; negative likelihood ratio: 0.2)<br>A value FLI between 60 and 100 can be used to rule-in SLD (specificity 86%; positive likelihood ratio: 4.3).
Hyperuricaemia (HU) [33]	Serum uric acid (SUA) levels ≥ 7.0 mg/dL (416 µmol/L) for both adult male and female populations, or being on urate-lowering therapy (ULT) (ICD-10-CM: E79; ICPC-2: T92)*<br>To convert from mg/dL to mmol/L, multiply by 0.05948
Coronary heart disease (CHD) [5,6]	Ischaemic heart disease (disease of the coronary arteries, usually from atherosclerosis, leading to myocardial ischaemia often associated with angina or myocardial infarction), myocardial infarction, coronary artery disease, acute coronary syndrome, acute and non-acute myocardial ischaemic syndromes, coronary revascularization (ICD-10-CM: I20-I25; ICPC-2: K74, K75, K76)*
Cerebrovascular disease (stroke) [7]	Cerebral ischaemia, ischaemic stroke, transient ischaemic attack, subarachnoid haemorrhage, intracerebral haemorrhage, intracranial haemorrhage (ICD-10-CM: I60-I66, I66, I67; ICPC-2: K89, K90K K91)*
Peripheral arterial disease (PAD) [8]	Lower-extremity peripheral arterial disease, intermittent claudication (fatigue, cramping, aching, pain, or other discomfort of vascular origin in the muscles of the lower extremities that is consistently induced by walking and consistently relieved by rest, usually within approximately 10 min), ankle-brachial index ≤ 0.9 (ICD-10-CM: I70.2, I73.9; ICPC-2: K92)*
Atherosclerotic cardiovascular disease (ASCVD)	ASCVD include CHD, stroke, or PAD (ICD-10-CM: I70)*

Heart failure (HF) [37]	Record of HF diagnosis (ICD-10-CM: I50; ICPC-2: K77)* in the primary care electronic health records, without differentiating by phenotype based on measurement of left ventricular ejection fraction or based on severity of symptoms and physical activity.
Atrial fibrillation (AF) [38,39]	Record of AF diagnosis (ICD-10-CM: I48; ICPC-2: K78)* in the primary care electronic health records, without differentiating by phenotypes based on paroxysmal, persistent, long-standing persistent, or permanent AF or atrial flutter.
Estimated glomerular filtration rate (eGFR) [18]	According to Chronic Kidney Disease Epidemiology Collaboration (CKD-EPI) equations:<br>Women with creatinine ≤ 0.7 mg/dL= 144 x $(\text{creatinine})^{-0.329}$ x $(0.993)^{\text{age}}$ mL/min/1.73 m^2 of the body surface<br>Women with creatinine > 0.7 mg/dL= 144 x $(\text{creatinine})^{-1.209}$ x $(0.993)^{\text{age}}$ mL/min/1.73 m^2 of the body surface<br>Men with creatinine ≤ 0.9 mg/dL= 141 x $(\text{creatinine})^{-0.411}$ x $(0.993)^{\text{age}}$ mL/min/1.73 m^2 of the body surface<br>Men with creatinine > 0.9 mg/dL= 141 x $(\text{creatinine})^{-1.209}$ x $(0.993)^{\text{age}}$ mL/min/1.73 m^2 of the body surface
Low eGFR [35]	eGFR < 60 mL/min/1.73 m^2 according to CKD-EPI [18]<br>Low eGFR does not include the following categories:<br>• G1: ≥ 90 mL/min/1.73 m^2<br>• G2: 60 to 89 mL/min/1.73 m^2<br>Low eGFR includes the following categories:<br>• G3a: 45 to 59 mL/min/1.73 m^2<br>• G3b: 30 to 44 mL/min/1.73 m^2<br>• G4: 15 to 29 mL/min/1.73 m^2<br>• G5: < 15 mL/min/1.73 m^2
Albuminuria [35]	Urine albumin-creatinine ratio (uACR) ≥ 30 mg/g (including proteinuria [uACR > 300 mg/g] (ICD-10-CM: R80; ICPC-2: U98)*<br>Albuminuria does not include the following category:<br>• A1: < 30 mg/g<br>Albuminuria includes the following categories:<br>• A2: 30 mg/g to 300 mg/g<br>• A3: > 300 mg/g<br>To convert from mg/g to mg/mmol, multiply by 0.01131
Chronic kidney disease (CKD) [35]	Low eGFR and/or albuminuria (ICD-10-CM: N18; ICPC-2: U99)*
CKD risk categories [35]	Risk of CKD progression, acute kidney injury, kidney failure replacement therapy, all-cause mortality and cardiovascular events.<br>• Low risk: G1A1; G2A1<br>• Moderate risk: G1A2; G2A2; G3aA1<br>• High risk: G1A3; G2A3; G3aA2; G3bA1<br>• Very high risk: G3aA3; G3bA2; G3bA3; G4A1; G4A2; G4A3; G5A1; G5A2; G5A3

Cardiovascular risk (CVR) categories [48]	Ten-year fatal and non-fatal cardiovascular disease (CVD) risk for patients from low-risk European countries.<br>Low, moderate, high and very high CVR categories were estimated according to 2021 ESC Guidelines on cardiovascular disease prevention in clinical practice.
Cardiovascular-kidney-metabolic (CKM) syndrome [9,10]	Systemic disorder attributable to pathophysiological interactions among metabolic risk factors, CKD, and CVD, that includes both individuals at risk for CVD, CKD, and those with existing clinical CVD.<br>• Stage 0: BMI < 25 kg/m^2, normal abdominal circumference (< 88 in women and < 102 cm in men) without criteria for the other stages.<br>• Stage 1: adiposity excess (CUN-BAE obesity), overweight, obesity, abdominal obesity, or prediabetes.<br>• Stage 2: HTN, metabolic risk factors (HTG, DM, MetS), CKD moderate or high risk.<br>• Stage 3: subclinical target organ damage, risk equivalents (high CVR or CKD very high-risk) among individuals with stages 1 or 2.<br>• Stage 4: clinical CVD including CHD, stroke, PAD, HF, and AF among individuals with stages 1 or 2 (stage 4a: without CKD; stage 4b: with CKD).<br>Stages 3 or 4 are defined as advanced stages of CKM syndrome because they identify individuals at high risk for CVD.

* ICD-10CM and ICPC-2 codes:

14. National Center for Health Statistics (NCHS). International Classification of Diseases, Tenth Revision, Clinical Modification (ICD-10-CM). Available online: https://www.cdc.gov/nchs/icd/icd-10-cm/index.html. (accessed 5 May 2025).

15. World Health Organization. (2009). International Classification of Primary Care, 2nd edition -ICPC-2. Available online: https://www.who.int/standards/classifications/other-classifications/international-classification-of-primary-care. (accessed 5 May 2025).

All citations in this table are included in the references section of the main manuscript.

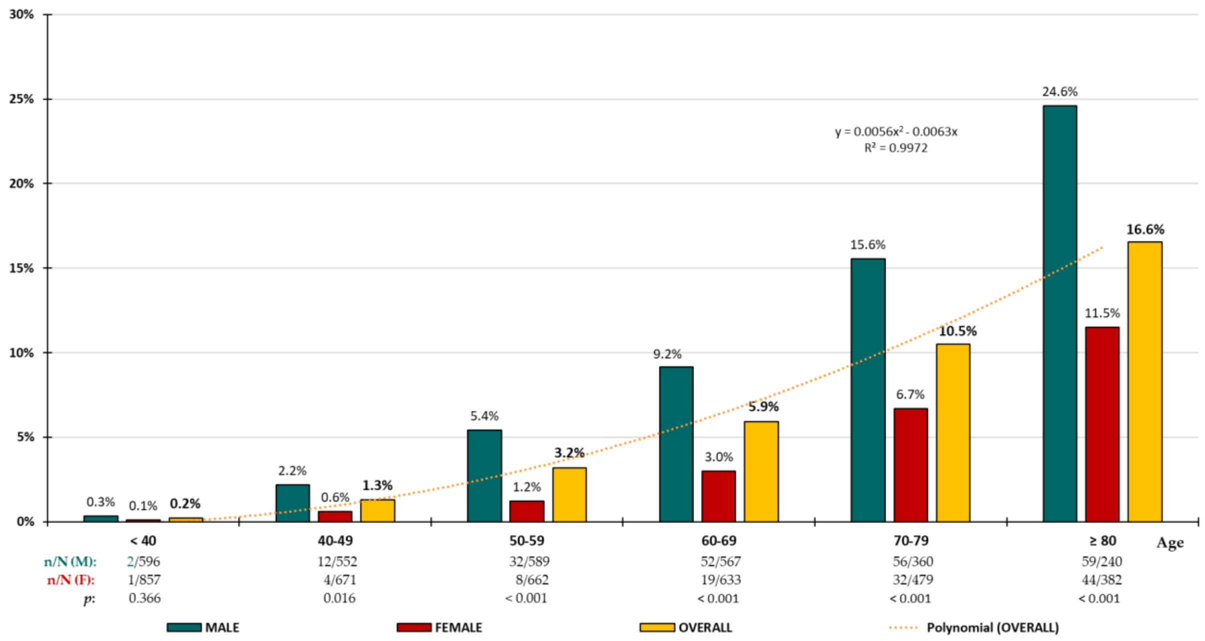

Figure S1. Age-Specific Prevalence Rates of Coronary Heart Disease

n: number of cases; N: sample size; M: male; F: female; *p*: *p*-value of the difference in percentages (M *vs.* F).

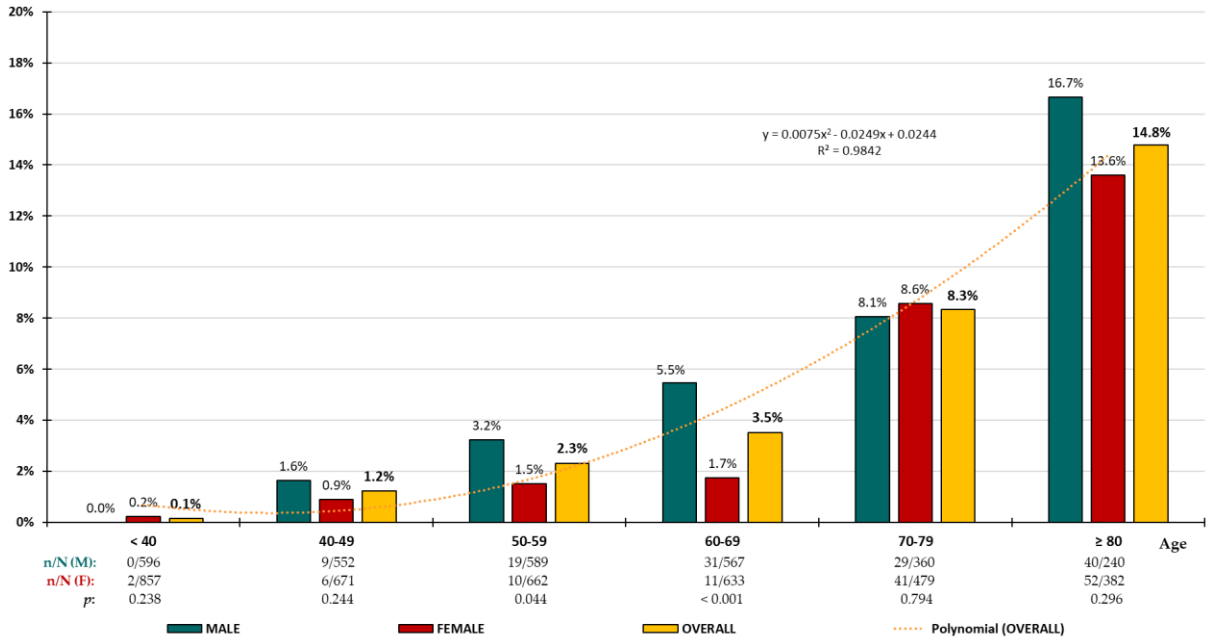

(b)

Figure S2. Age-Specific Prevalence Rates of Stroke

n: number of cases; N: sample size; M: male; F: female; *p*: *p*-value of the difference in percentages (M *vs.* F).

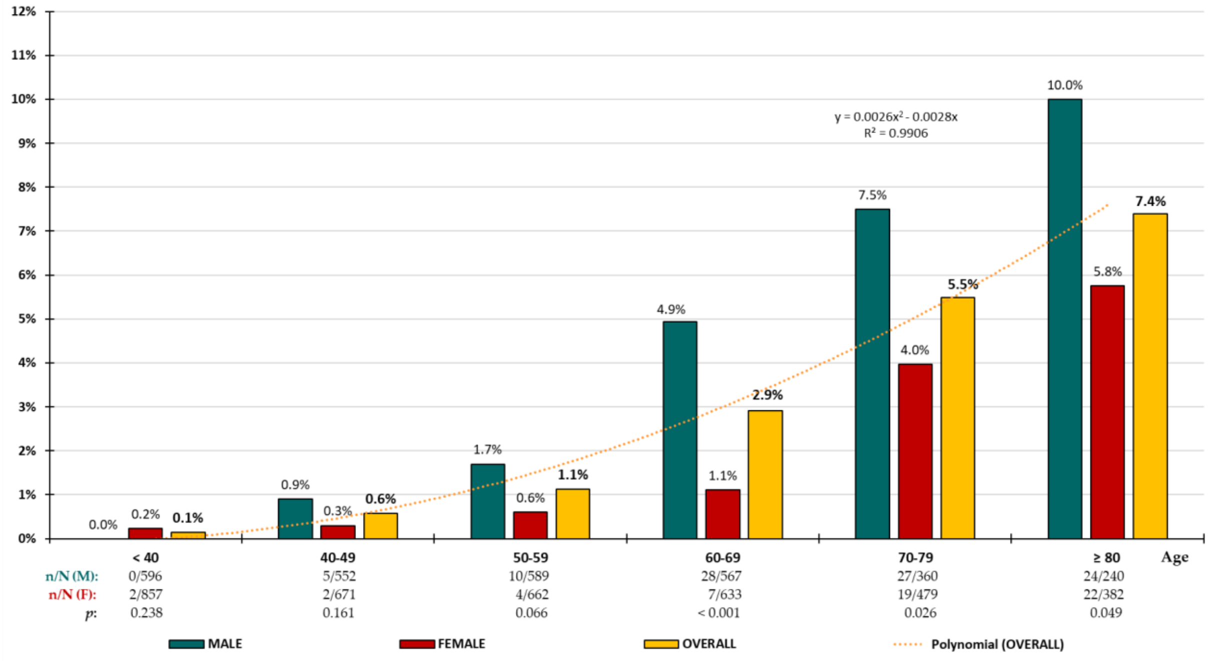

Figure S3. Age-Specific Prevalence Rates of Peripheral Arterial Disease

n: number of cases; N: sample size; M: male; F: female; *p*: *p*-value of the difference in percentages (M *vs.* F).

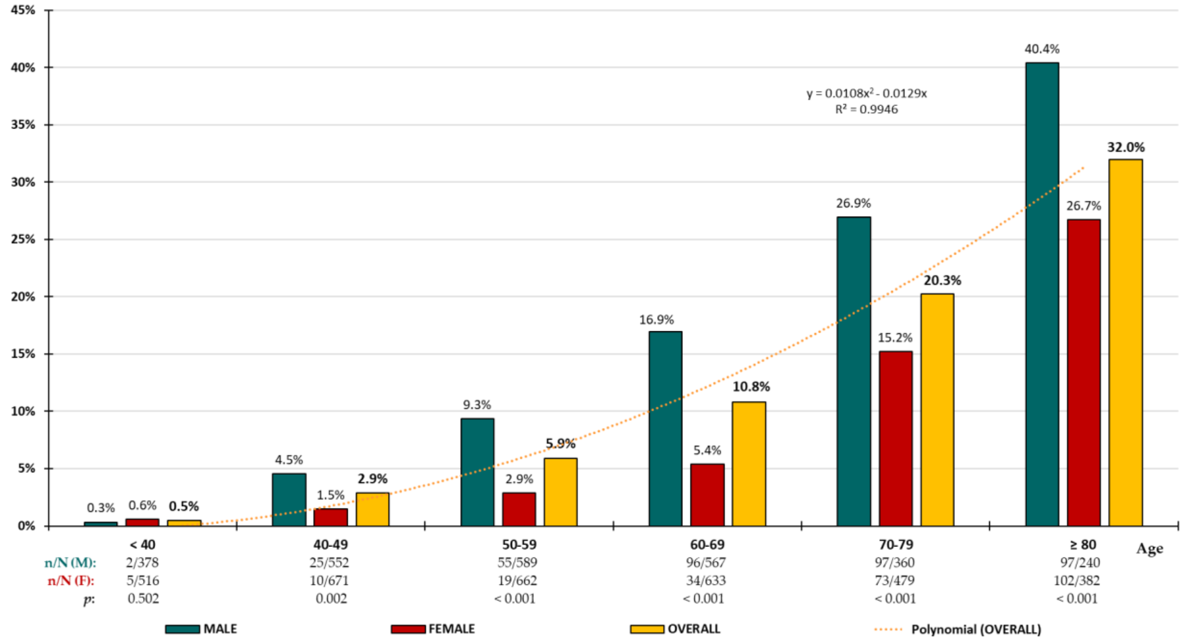

Figure S4. Age-Specific Prevalence Rates of Atherosclerotic Cardiovascular Disease

n: number of cases; N: sample size; M: male; F: female; *p*: *p*-value of the difference in percentages (M *vs.* F).

Table S2. Clinical characteristics for populations with ASCVDs

	**CHD*** Mean (SD)	**Stroke*** Mean (SD)	**PAD*** Mean (SD)	**ASCVD*** Mean (SD)
Age yr	72.1 (12.8)	73.3 (13.4)	72.6 (12.1)	72.1 (13.1)
BMI kg/m^2	29.6 (5.4)	29.0 (5.1)	28.7 (4.7)	29.2 (5.2)
WC cm	100.5 (13.4)	97.8 (12.9)	98.2 (12.4)	99.0 (13.2)
WtHR	0.62 (0.08)	0.61 (0.08)	0.61 (0.08)	0.61 (0.08)
CUN-BAE adiposity	36.6 (8.0)	37.8 (8.1)	36.1 (7.7)	36.8 (8.0)
VAI	1.99 (1.47)	1.93 (1.40)	1.94 (1.27)	1.97 (1.45)
BSI	0.083 (0.01)	0.082 (0.01)	0.082 (0.01)	0.082 (0.01)
BRI	5.95 (1.97)	5.79 (1.90)	5.66 (1.78)	5.82 (1.94)
LAP index	57.1 (43.2)	53.2 (39.4)	53.7 (34.4)	55.4 (41.7)
SBP mmHg	127.0 (15.5)	128.1 (15.3)	128.3 (15.2)	127.5 (15.3)
DBP mmHg	73.3 (9.8)	74.1 (9.7)	73.0 (9.0)	73.7 (9.6)
FPG mg/dL	110.3 (32.6)	104.7 (27.6)	107.6 (33.3)	108.0 (32.0)
HbA1c %	6.20 (1.04)	5.98 (0.96)	6.18 (0.98)	6.11 (1.02)
TC mg/dL	163.8 (37.6)	180.6 (41.1)	170.1 (39.3)	173.0 (40.1)
HDL-c mg/dL	50.0 (14.9)	52.1 (13.7)	50.5 (13.5)	51.0 (14.1)
LDL-c mg/dL	88.2 (31.0)	103.8 (35.4)	93.2 (31.9)	96.5 (34.0)
TG mg/dL	128.8 (70.4)	125.5 (71.5)	131.3 (69.2)	128.6 (72.7)
TG/HDL-c	2.96 (2.24)	2.70 (2.02)	2.86 (1.92)	2.87 (2.19)
AIP	0.02 (0.28)	−0.02 (0.27)	0.02 (0.25)	0.00 (0.28)
TyG index	8.72 (0.60)	8.64 (0.59)	8.71 (0.60)	8.69 (0.60)
SUA mg/dL	5.53 (1.51)	5.44 (1.59)	5.51 (1.49)	5.48 (1.56)
AST U/L	25.5 (40.4)	21.5 (12.4)	22.2 (17.9)	24.2 (31.4)
ALT U/L	26.6 (17.1)	24.0 (24.4)	26.3 (17.9)	26.2 (21.3)
GGT U/L	44.2 (53.5)	39.8 (48.0)	43.7 (43.6)	41.6 (48.5)
FLI 0–100	60.5 (27.1)	55.3 (27.0)	57.8 (26.5)	57.7 (27.3)
Creatinine mg/dL	1.02 (0.48)	0.93 (0.30)	1.07 (0.65)	0.98 (0.44)
eGFR mL/min/1.73 m^2	72.5 (20.6)	74.4 (21.0)	70.0 (21.5)	73.7 (20.7)
uACR mg/g	39.4 (139.7)	37.2 (119.6)	53.0 (124.0)	40.1 (129.5)

AIP: atherogenic index of plasma; ALT: alanine aminotransferase; ASCVD: atherosclerotic cardiovascular disease; AST: aspartate aminotransferase; BMI: body mass index; BRI: body roundness index; BSI: body shape index; CHD: coronary heart disease; CI: confidence interval; CUN-BAE: according to its acronym in Spanish, *Clínica Universitaria de Navarra* - Body Adiposity Estimator; DBP: diastolic blood pressure; eGFR: estimated glomerular filtration rate; FPG: fasting plasma glucose; FLI: fatty liver index; GGT: gamma-glutamyl transferase; HbA1c: glycated haemoglobin A1c; HDL-c: high-density lipoprotein cholesterol; LAP: lipid accumulation product index; LDL-c: low-density lipoprotein cholesterol; PAD: peripheral arterial disease; SBP: systolic blood pressure; SD: standard deviation; SUA: serum uric acid; TC: total cholesterol; TG: triglyceride; TyG: triglyceride-glucose index; uACR: urine albumin-creatinine ratio; VAI: visceral adiposity index; WC: waist circumference; WtHR: waist-to-height ratio. * No. CHD: 321; No. stroke: 250; No. PAD: 150; No. ASCVD: 615

Table S3. Quantitative clinical variables in populations with and without CHD

	With CHD No. 321	Without CHD No. 6267	With CHD *vs.* without CHD	
	**Mean (SD)**	**Mean (SD)**	***p***	**Cohen's *d* (95% CI)**
Age yr	72.1 (12.8)	54.3 (17.3)	< 0.001	1.0 (0.9; 1.2)
Male age yr	70.6 (12.4)	54.0 (16.6)	< 0.001	1.0 (0.9; 1.2)
Female age yr	75.1 (13.2)	54.4 (17.8)	< 0.001	1.2 (1.0; 1.4)
BMI kg/m^2	29.6 (5.4)	27.4 (5.1)	< 0.001	0.4 (0.3; 0.5)
WC cm	100.5 (13.4)	93.0 (14.0)	< 0.001	0.5 (0.4; 0.7)
WtHR	0.62 (0.08)	0.57 (0.09)	< 0.001	0.6 (0.5; 0.7)
CUN-BAE adiposity	36.6 (8.0)	34.6 (8.7)	< 0.001	0.2 (0.1; 0.3)
VAI	1.99 (1.47)	1.78 (1.70)	0.027	0.2 (0.0; 0.2)
BSI	0.083 (0.01)	0.080 (0.01)	< 0.001	0.2 (0.1; 0.3)
BRI	5.95 (1.97)	4.87 (1.96)	< 0.001	0.6 (0.4; 0.7)
LAP index	57.1 (43.2)	45.9 (40.5)	< 0.001	0.3 (0.2; 0.4)
SBP mmHg	127.0 (15.5)	121.7 (15.4)	< 0.001	0.3 (0.2; 0.5)
DBP mmHg	73.3 (9.8)	73.3 (9.8)	0.988	0.0 (−0.1; 0.1)
FPG mg/dL [a]	110.3 (32.6)	95.3 (25.3)	< 0.001	0.6 (0.5; 0.7)
HbA1c % [b] #	6.20 (1.04)	5.60 (0.88)	< 0.001	0.7 (0.5; 0.7)
TC mg/dL [c]	163.8 (37.6)	194.3 (38.9)	< 0.001	−0.8 (−0.9; −0.7)
HDL-c mg/dL [c]	50.0 (14.9)	55.1 (14.6)	< 0.001	−0.4 (−0.5; −0.2)
LDL-c mg/dL # [c]	88.2 (31.0)	115.5 (34.2)	< 0.001	−0.8 (−0.9; −0.7)
TG mg/dL [d]	128.8 (70.4)	120.1 (83.8)	0.068	0.1 (0.0; 0.2)
TG/HDL-c	2.96 (2.24)	2.51 (2.57)	0.002	0.2 (0.1; 0.3)
AIP	0.02 (0.28)	−0.07 (0.29)	< 0.001	0.3 (0.2; 0.4)
TyG index	8.72 (0.60)	8.48 (0.60)	< 0.001	0.4 (0.3; 0.5)
SUA mg/dL # [e]	5.53 (1.51)	4.94 (1.47)	< 0.001	0.4 (0.3; 0.5)
AST U/L #	25.5 (40.4)	22.9 (43.3)	0.361	0.1 (−0.1; 0.2)
ALT U/L #	26.6 (17.1)	24.8 (16.9)	0.060	0.1 (0.0; 0.2)
GGT U/L #	44.2 (53.5)	32.9 (50.6)	< 0.001	0.2 (0.1; 0.3)
FLI 0–100 #	60.5 (27.1)	44.3 (30.5)	< 0.001	0.5 (0.4; 0.7)
Creatinine mg/dL [f]	1.02 (0.48)	0.83 (0.28)	< 0.001	0.7 (0.5; 0.8)
eGFR mL/min/1.73 m^2	72.5 (20.6)	91.5 (20.1)	< 0.001	−0.9 (−1.1; −0.8)
uACR mg/g [g]	39.4 (139.7)	15.2 (53.1)	< 0.001	0.4 (0.3; 0.5)

CHD: coronary heart disease; CI: confidence interval; Cohen's *d*; effect size of standardised mean difference according to the proximity to the following absolute *d*-values: 0.2 small; 0.5 medium; 0.8 large; *p*: *p*-value of the difference in means; SD: standard deviation.

AIP: atherogenic index of plasma; ALT: alanine aminotransferase (# No. with CHD: 317, without CHD: 6105); AST: aspartate aminotransferase (# No. with CHD: 243, without CHD: 4578); BMI: body mass index; BRI: body roundness index; BSI: body shape index; CUN-BAE: according to its acronym in Spanish, *Clínica Universitaria de Navarra* - Body Adiposity Estimator; DBP: diastolic blood pressure; eGFR: estimated glomerular filtration rate; FPG: fasting plasma glucose; FLI: fatty liver index (# No. with CHD: 299, without CHD: 5809); GGT: gamma-glutamyl transferase (# No. with CHD: 299, without CHD: 5809); HbA1c: glycated haemoglobin A1c (# No. with CHD: 290, without CHD: 4943); HDL-c: high-density lipoprotein cholesterol; LAP: lipid accumulation product index; LDL-c: low-density lipoprotein cholesterol (# No. with CHD: 318, without CHD: 6208); SBP: systolic blood pressure; SUA: serum uric acid (# No. with CHD: 315, without CHD: 6174); TC: total cholesterol; TG: triglyceride; TyG: triglyceride-glucose index; uACR: urine albumin-creatinine ratio; VAI: visceral adiposity index; WC: waist circumference; WtHR: waist-to-height ratio.

[a] To convert from mg/dL to mmol/L, multiply by 0.05556

[b] To convert from % (DCCT) to mmol/mol (IFCC), subtract 2.15 and multiply by 10.929

[c] To convert from mg/dL to mmol/L, multiply by 0.02586

[d] To convert from mg/dL to mmol/L, multiply by 0.01129

[e] To convert from mg/dL to mmol/L, multiply by 0.05948

[f] To convert from mg/dL to mmol/L, multiply by 0.08842

[g] To convert from mg/g to mg/mmol, multiply by 0.01131

Table S4. Quantitative clinical variables in populations with and without Stroke

	**With stroke** No. 250	**Without stroke** No. 6338	**With stroke *vs.* without stroke**	
	**Mean** (SD)	**Mean** (SD)	***p***	**Cohen's *d*** (95% CI)
Age yr	73.3 (13.4)	54.4 (17.3)	< 0.001	1.1 (1.0; 1.2)
Male age yr	71.2 (13.2)	54.5 (16.6)	< 0.001	1.0 (0.8; 1.2)
Female age yr	75.5 (13.3)	54.4 (17.8)	< 0.001	1.2 (1.0; 1.4)
BMI kg/m^2	29.0 (5.1)	27.5 (5.1)	< 0.001	0.3 (0.2; 0.4)
WC cm	97.8 (12.9)	93.2 (14.1)	< 0.001	0.3 (0.2; 0.5)
WtHR	0.61 (0.08)	0.57 (0.09)	< 0.001	0.5 (0.3; 0.6)
CUN-BAE adiposity	37.8 (8.1)	34.6 (8.7)	< 0.001	0.4 (0.2; 0.5)
VAI	1.93 (1.40)	1.78 (1.70)	0.163	0.1 (0.0; 0.2)
BSI	0.082 (0.01)	0.080 (0.01)	< 0.001	0.2 (0.1; 0.3)
BRI	5.79 (1.90)	4.89 (1.97)	< 0.001	0.5 (0.3; 0.6)
LAP index	53.2 (39.4)	46.2 (40.7)	0.008	0.2 (0.0; 0.3)
SBP mmHg	128.1 (15.3)	121.7 (15.4)	< 0.001	0.4 (0.3; 0.5)
DBP mmHg	74.1 (9.7)	73.3 (9.8)	0.208	0.1 (0.0; 0.2)
FPG mg/dL [a]	104.7 (27.6)	95.7 (25.8)	< 0.001	0.4 (0.2; 0.5)
HbA1c % [b] #	5.98 (0.96)	5.62 (0.89)	< 0.001	0.4 (0.3; 0.5)
TC mg/dL [c]	180.6 (41.1)	193.3 (39.2)	< 0.001	−0.3 (−0.5; −0.2)
HDL-c mg/dL [c]	52.1 (13.7)	54.9 (14.7)	0.002	−0.2 (−0.3; −0.1)
LDL-c mg/dL # [c]	103.8 (35.4)	114.6 (34.4)	< 0.001	−0.3 (−0.4; −0.2)
TG mg/dL [d]	125.5 (71.5)	120.3 (83.6)	0.336	0.1 (−0.1; 0.2)
TG/HDL-c	2.70 (2.02)	2.52 (2.57)	0.276	0.1 (−0.1; 0.2)
AIP	−0.02 (0.27)	−0.07 (0.29)	0.006	0.2 (0.1; 0.3)
TyG index	8.64 (0.59)	8.49 (0.61)	< 0.001	0.3 (0.1; 0.4)
SUA mg/dL # [e]	5.44 (1.59)	4.95 (1.47)	< 0.001	0.3 (0.2; 0.5)
AST U/L #	21.5 (12.4)	23.1 (43.9)	0.609	0.0 (−0.2; 0.1)
ALT U/L #	24.0 (24.4)	24.9 (16.6)	0.427	−0.1 (−0.2; 0.1)
GGT U/L #	39.8 (48.0)	33.2 (50.9)	0.048	0.1 (0.0; 0.3)
FLI 0–100 #	55.3 (27.0)	44.7 (30.6)	< 0.001	0.4 (0.2; 0.5)
Creatinine mg/dL [f]	0.93 (0.30)	0.84 (0.29)	< 0.001	0.3 (0.2; 0.4)
eGFR mL/min/1.73 m^2	74.4 (21.0)	91.2 (20.3)	< 0.001	−0.8 (−1.0; −0.7)
uACR mg/g [g]	37.2 (119.6)	15.6 (56.7)	< 0.001	0.4 (0.2; 0.5)

CI: confidence interval; Cohen's *d*; effect size of standardised mean difference according to the proximity to the following absolute *d*-values: 0.2 small; 0.5 medium; 0.8 large; *p*: *p*-value of the difference in means; SD: standard deviation.

AIP: atherogenic index of plasma; ALT: alanine aminotransferase (# No. with stroke: 246, without stroke: 6176); AST: aspartate aminotransferase (# No. with stroke: 187, without stroke: 4634); BMI: body mass index; BRI: body roundness index; BSI: body shape index; CHD: coronary heart disease; CUN-BAE: according to its acronym in Spanish, *Clínica Universitaria de Navarra* - Body Adiposity Estimator; DBP: diastolic blood pressure; eGFR: estimated glomerular filtration rate; FPG: fasting plasma glucose; FLI: fatty liver index (# No. with stroke: 237, without stroke: 5871); GGT: gamma-glutamyl transferase (# No. with stroke: 237, without stroke: 5871); HbA1c: glycated haemoglobin A1c (# No. with stroke: 229, without stroke: 5004); HDL-c: high-density lipoprotein cholesterol; LAP: lipid accumulation product index; LDL-c: low-density lipoprotein cholesterol (# No. with stroke: 248, without stroke: 6278); SBP: systolic blood pressure; SUA: serum uric acid (# No. with stroke: 247, without stroke: 6242); TC: total cholesterol; TG: triglyceride; TyG: triglyceride-glucose index; uACR: urine albumin-creatinine ratio; VAI: visceral adiposity index; WC: waist circumference; WtHR: waist-to-height ratio.

[a] To convert from mg/dL to mmol/L, multiply by 0.05556

[b] To convert from % (DCCT) to mmol/mol (IFCC), subtract 2.15 and multiply by 10.929

[c] To convert from mg/dL to mmol/L, multiply by 0.02586

[d] To convert from mg/dL to mmol/L, multiply by 0.01129

[e] To convert from mg/dL to mmol/L, multiply by 0.05948

[f] To convert from mg/dL to mmol/L, multiply by 0.08842

[g] To convert from mg/g to mg/mmol, multiply by 0.01131

Table S5. Quantitative clinical variables in populations with and without PAD

	**With PAD** No. 150	**Without PAD** No. 6438	**With PAD *vs.* without PAD**	
	**Mean** (SD)	**Mean** (SD)	***p***	**Cohen's *d*** (95% CI)
Age yr	72.6 (12.1)	54.7 (17.4)	< 0.001	1.0 (0.9; 1.2)
Male age yr	71.6 (11.1)	54.7 (16.4)	< 0.001	1.0 (0.9; 1.2)
Female age yr	74.4 (13.6)	54.8 (17.9)	< 0.001	1.1 (0.8; 1.4)
BMI kg/m^2	28.7 (4.7)	27.5 (5.2)	0.004	0.2 (0.1; 0.4)
WC cm	98.2 (12.4)	93.2 (14.1)	< 0.001	0.4 (0.2; 0.5)
WtHR	0.61 (0.08)	0.57 (0.09)	< 0.001	0.5 (0.3; 0.6)
CUN-BAE adiposity	36.1 (7.7)	34.7 (8.7)	0.048	0.2 (0.0; 0.3)
VAI	1.94 (1.27)	1.78 (1.70)	0.253	0.1 (−0.1; 0.3)
BSI	0.082 (0.01)	0.080 (0.01)	< 0.001	0.2 (0.1; 0.3)
BRI	5.66 (1.78)	4.90 (1.97)	< 0.001	0.4 (0.2; 0.6)
LAP index	53.7 (34.4)	46.3 (40.8)	0.026	0.2 (0.0; 0.3)
SBP mmHg	128.3 (15.2)	121.8 (15.4)	< 0.001	0.4 (0.3; 0.6)
DBP mmHg	73.0 (9.0)	73.3 (9.8)	0.680	0.0 (−0.2; 0.1)
FPG mg/dL [a]	107.6 (33.3)	95.8 (25.7)	< 0.001	0.5 (0.3; 0.6)
HbA1c % [b] #	6.18 (0.98)	5.62 (0.89)	< 0.001	0.6 (0.5; 0.8)
TC mg/dL [c]	170.1 (39.3)	193.3 (39.2)	< 0.001	−0.6 (−0.8; −0.4)
HDL-c mg/dL [c]	50.5 (13.5)	54.9 (14.7)	< 0.001	−0.3 (−0.5; −0.1)
LDL-c mg/dL # [c]	93.2 (31.9)	114.6 (34.4)	< 0.001	−0.6 (−0.8; −0.5)
TG mg/dL [d]	131.3 (69.2)	120.3 (83.5)	0.107	0.1 (0.0; 0.3)
TG/HDL-c	2.86 (1.92)	2.52 (2.57)	0.112	0.1 (0.0; 0.3)
AIP	0.02 (0.25)	−0.07 (0.29)	< 0.001	0.3 (0.2; 0.5)
TyG index	8.71 (0.60)	8.49 (0.60)	< 0.001	0.3 (0.3; 0.4)
SUA mg/dL # [e]	5.51 (1.49)	4.95 (1.48)	< 0.001	0.4 (0.2; 0.5)
AST U/L #	22.2 (17.9)	23.1 (43.6)	0.837	0.0 (−0.2; 0.2)
ALT U/L #	26.3 (17.9)	24.8 (16.9)	0.313	0.1 (−0.1; 0.3)
GGT U/L #	43.7 (43.6)	33.2 (50.9)	0.014	0.2 (0.0; 0.4)
FLI 0–100 #	57.8 (26.5)	44.8 (30.6)	< 0.001	0.4 (0.3; 0.6)
Creatinine mg/dL [f]	1.07 (0.65)	0.84 (0.28)	< 0.001	0.8 (0.6; 1.0)
eGFR mL/min/1.73 m^2	70.0 (21.5)	91.0 (20.3)	< 0.001	−1.0 (−1.2; −0.9)
uACR mg/g [g]	53.0 (124.0)	15.6 (57.9)	< 0.001	0.6 (0.5; 0.8)

PAD: peripheral arterial disease; CI: confidence interval; Cohen's *d*; effect size of standardised mean difference according to the proximity to the following absolute *d*-values: 0.2 small; 0.5 medium; 0.8 large; *p*: *p*-value of the difference in means; SD: standard deviation.

AIP: atherogenic index of plasma; ALT: alanine aminotransferase (# No. with PAD: 148, without PAD: 6274); AST: aspartate aminotransferase (# No. with PAD: 109, without PAD: 4712); BMI: body mass index; BRI: body roundness index; BSI: body shape index; CHD: coronary heart disease; CUN-BAE: according to its acronym in Spanish, *Clínica Universitaria de Navarra* - Body Adiposity Estimator; DBP: diastolic blood pressure; eGFR: estimated glomerular filtration rate; FPG: fasting plasma glucose; FLI: fatty liver index (# No. with PAD: 144, without PAD: 5964); GGT: gamma-glutamyl transferase (# No. with PAD: 144, without PAD: 5964); HbA1c: glycated haemoglobin A1c (# No. with PAD: 139, without PAD: 5094); HDL-c: high-density lipoprotein cholesterol; LAP: lipid accumulation product index; LDL-c: low-density lipoprotein cholesterol (# No. with PAD: 149, without PAD: 6377); SBP: systolic blood pressure; SUA: serum uric acid (# No. with PAD: 148, without PAD: 6341); TC: total cholesterol; TG: triglyceride; TyG: triglyceride-glucose index; uACR: urine albumin-creatinine ratio; VAI: visceral adiposity index; WC: waist circumference; WtHR: waist-to-height ratio.

[a] To convert from mg/dL to mmol/L, multiply by 0.05556

[b] To convert from % (DCCT) to mmol/mol (IFCC), subtract 2.15 and multiply by 10.929

[c] To convert from mg/dL to mmol/L, multiply by 0.02586

[d] To convert from mg/dL to mmol/L, multiply by 0.01129

[e] To convert from mg/dL to mmol/L, multiply by 0.05948

[f] To convert from mg/dL to mmol/L, multiply by 0.08842

[g] To convert from mg/g to mg/mmol, multiply by 0.01131

Table S6. Quantitative clinical variables in populations with and without ASCVD

	**With ASCVD** No. 615	**Without ASCVD** No. 5973	**With ASCVD *vs.* without ASCVD**	
	**Mean** (SD)	**Mean** (SD)	***p***	**Cohen's *d*** (95% CI)
Age yr	72.1 (13.1)	53.4 (17.0)	< 0.001	1.1 (1.0; 1.2)
Male age yr	70.2 (12.4)	53.1 (16.3)	< 0.001	1.1 (1.0; 1.2)
Female age yr	74.9 (13.5)	53.7 (17.5)	< 0.001	1.2 (1.1; 1.4)
BMI kg/m^2	29.2 (5.2)	27.3 (5.1)	< 0.001	0.4 (0.3; 0.5)
WC cm	99.0 (13.2)	92.8 (14.0)	< 0.001	0.5 (0.4; 0.5)
WtHR	0.61 (0.08)	0.57 (0.09)	< 0.001	0.5 (0.4; 0.6)
CUN-BAE adiposity	36.8 (8.0)	34.5 (8.7)	< 0.001	0.3 (0.2; 0.4)
VAI	1.97 (1.45)	1.77 (1.71)	0.006	0.1 (0.0; 0.2)
BSI	0.082 (0.01)	0.080 (0.01)	< 0.001	0.2 (0.1; 0.3)
BRI	5.82 (1.94)	4.83 (1.95)	< 0.001	0.5 (0.4; 0.6)
LAP index	55.4 (41.7)	45.5 (40.5)	< 0.001	0.2 (0.2; 0.3)
SBP mmHg	127.5 (15.3)	121.3 (15.3)	< 0.001	0.4 (0.3; 0.5)
DBP mmHg	73.7 (9.6)	73.3 (9.8)	0.319	0.0 (0.0; 0.1)
FPG mg/dL [a]	108.0 (32.0)	94.8 (24.9)	< 0.001	0.5 (0.4; 0.6)
HbA1c % [b] #	6.11 (1.02)	5.58 (0.87)	< 0.001	0.6 (0.5; 0.7)
TC mg/dL [c]	173.0 (40.1)	194.8 (38.7)	< 0.001	−0.6 (−0.6; −0.5)
HDL-c mg/dL [c]	51.0 (14.1)	55.2 (14.7)	< 0.001	−0.3 (−0.4; −0.2)
LDL-c mg/dL # [c]	96.5 (34.0)	116.0 (34.0)	< 0.001	−0.6 (−0.7; −0.5)
TG mg/dL [d]	128.6 (72.7)	119.7 (84.2)	0.012	0.1 (0.0; 0.2)
TG/HDL-c	2.87 (2.19)	2.49 (2.59)	0.001	0.2 (0.1; 0.2)
AIP	0.00 (0.28)	−0.07 (0.29)	< 0.001	0.2 (0.2; 0.3)
TyG index	8.69 (0.60)	8.47 (0.60)	< 0.001	0.4 (0.3; 0.5)
SUA mg/dL # [e]	5.48 (1.56)	4.91 (1.46)	< 0.001	0.4 (0.3; 0.5)
AST U/L #	24.2 (31.4)	23.0 (44.2)	0.566	0.0 (0.0; 0.1)
ALT U/L #	26.2 (21.3)	24.7 (16.4)	0.045	0.1 (0.0; 0.2)
GGT U/L #	41.6 (48.5)	32.6 (50.9)	< 0.001	0.2 (0.1; 0.3)
FLI 0–100 #	57.7 (27.3)	43.8 (30.5)	< 0.001	0.5 (0.4; 0.6)
Creatinine mg/dL [f]	0.98 (0.44)	0.83 (0.27)	< 0.001	0.5 (0.4; 0.6)
eGFR mL/min/1.73 m^2	73.7 (20.7)	92.3 (19.7)	< 0.001	−0.9 (−1.0; −0.9)
uACR mg/g [g]	40.1 (129.5)	14.0 (47.3)	< 0.001	0.4 (0.4; 0.5)

ASCVD: atherosclerotic cardiovascular disease; CI: confidence interval; Cohen's *d*; effect size of standardised mean difference according to the proximity to the following absolute *d*-values: 0.2 small; 0.5 medium; 0.8 large; *p*: *p*-value of the difference in means; SD: standard deviation.

AIP: atherogenic index of plasma; ALT: alanine aminotransferase (# No. with ASCVD: 605, without ASCVD: 5817); AST: aspartate aminotransferase (# No. with ASCVD: 462, without ASCVD: 4359); BMI: body mass index; BRI: body roundness index; BSI: body shape index; CUN-BAE: according to its acronym in Spanish, *Clínica Universitaria de Navarra* - Body Adiposity Estimator; DBP: diastolic blood pressure; eGFR: estimated glomerular filtration rate; FPG: fasting plasma glucose; FLI: fatty liver index (# No. with ASCVD: 580, without ASCVD: 5528); GGT: gamma-glutamyl transferase (# No. with ASCVD: 580, without ASCVD: 5528); HbA1c: glycated haemoglobin A1c (# No. with ASCVD: 558, without ASCVD: 4675); HDL-c: high-density lipoprotein cholesterol; LAP: lipid accumulation product index; LDL-c: low-density lipoprotein cholesterol (# No. with ASCVD: 609, without ASCVD: 5917); SBP: systolic blood pressure; SUA: serum uric acid (# No. with ASCVD: 605, without ASCVD: 5884); TC: total cholesterol; TG: triglyceride; TyG: triglyceride-glucose index; uACR: urine albumin-creatinine ratio; VAI: visceral adiposity index; WC: waist circumference; WtHR: waist-to-height ratio.

[a] To convert from mg/dL to mmol/L, multiply by 0.05556

[b] To convert from % (DCCT) to mmol/mol (IFCC), subtract 2.15 and multiply by 10.929

[c] To convert from mg/dL to mmol/L, multiply by 0.02586

[d] To convert from mg/dL to mmol/L, multiply by 0.01129

[e] To convert from mg/dL to mmol/L, multiply by 0.05948

[f] To convert from mg/dL to mmol/L, multiply by 0.08842

[g] To convert from mg/g to mg/mmol, multiply by 0.01131

Table S7. Diseases and medical conditions in populations with and without CHD

	With CHD N= 321 No. (%)	Without CHD N= 6267 No. (%)	With CHD *vs.* without CHD	
			p	OR (95% CI)
Male	213 (66.4)	2691 (42.9)	< 0.001	2.6 (2.1–3.3)
Current smoking	43 (13.4)	1383 (22.1)	< 0.001	0.6 (0.4–0.8)
Alcoholism	30 (9.3)	580 (9.3)	0.956	1.0 (0.7–1.5)
Physical inactivity	159 (49.5)	2920 (46.6)	0.303	1.1 (0.9–1.4)
Overweight	124 (38.6)	2392 (38.2)	0.868	1.0 (0.8–1.3)
Obesity	136 (42.4)	1697 (27.1)	< 0.001	2.0 (1.6–2.5)
Abdominal obesity	187 (58.3)	2735 (43.6)	< 0.001	1.8 (1.4–2.3)
High-WtHR	187 (58.3)	2174 (34.7)	< 0.001	2.6 (2.1–3.3)
CUN-BAE excess adiposity	302 (94.1)	4530 (72.3)	< 0.001	6.1 (3.8–9.7)
High-VAI	71 (22.1)	1045 (16.7)	0.011	1.4 (1.1–1.9)
High-BSI	151 (47.0)	2018 (32.2)	< 0.001	1.9 (1.5–2.3)
High-BRI	163 (50.8)	1791 (28.6)	< 0.001	2.6 (2.1–3.2)
High-LAP index	124 (38.6)	1687 (26.9)	< 0.001	1.7 (1.4–2.2)
Prediabetes	93 (29.0)	1356 (21.6)	0.002	1.5 (1.2–1.9)
Diabetes	137 (42.7)	899 (14.3)	< 0.001	4.4 (3.5–5.6)
Hypertension	299 (93.1)	2387 (38.1)	< 0.001	22.1 (14.3–34.2)
Hypercholesterolaemia	296 (92.2)	3836 (61.2)	< 0.001	7.5 (5.0–11.3)
Low HDL-c	136 (42.4)	1683 (26.9)	< 0.001	2.0 (1.6–2.5)
Hypertriglyceridaemia	136 (42.4)	1811 (28.9)	< 0.001	1.8 (1.4–2.3)
Atherogenic dyslipidaemia	80 (24.9)	861 (13.7)	< 0.001	2.1 (1.6–2.7)
High-AIP	59 (18.4)	917 (14.6)	0.065	1.3 (1.0–1.8)
High-TyG index	146 (45.5)	1926 (30.7)	< 0.001	1.9 (1.5–2.4)
Metabolic syndrome	296 (92.2)	2555 (40.8)	< 0.001	17.2 (11.4–26.0)
Fatty liver index ≥ 60 [a]	160 (53.5)	1990 (34.3)	< 0.001	2.2 (1.7–2.8)
Hyperuricaemia [b]	60 (19.0)	680 (11.0)	< 0.001	1.9 (1.4–2.5)
Stroke	48 (15.0)	202 (3.2)	< 0.001	5.3 (3.8–7.4)
Peripheral arterial disease	37 (11.5)	115 (1.8)	< 0.001	7.0 (4.7–10.3)
Erectile dysfunction [c]	124 (58.2)	380 (14.1)	< 0.001	8.5 (6.3–11.4)
Heart failure	55 (17.1)	129 (2.1)	< 0.001	9.8 (7.0–13.8)
Atrial fibrillation	43 (13.4)	207 (3.3)	< 0.001	4.5 (3.2–6.4)
Albuminuria	56 (17.4)	338 (5.4)	< 0.001	3.7 (2.7–5.0)
Low eGFR	88 (27.4)	436 (7.0)	< 0.001	5.1 (3.9–6.6)
CKD	107 (33.3)	649 (10.4)	< 0.001	4.3 (3.4–5.5)
CKD low risk	214 (66.7)	5618 (89.6)	< 0.001	0.2 (0.2–0.3)
CKD moderate risk	54 (16.8)	436 (7.0)	< 0.001	2.7 (2.0–3.7)
CKD high risk	32 (10.0)	132 (2.1)	< 0.001	5.1 (3.4–7.7)
CKD very high risk	21 (6.5)	81 (1.3)	< 0.001	5.3 (3.3–8.8)
LLT	271 (84.4)	1581 (25.2)	< 0.001	16.1 (11.8–21.8)
BPLT	289 (90.0)	2050 (32.7)	< 0.001	18.6 (12.8–26.9)
GLT	111 (34.6)	736 (11.7)	< 0.001	4.0 (3.1–5.1)

CHD: coronary heart disease; No. (%): cases number (percentage); CI: confidence interval; OR: odds ratio; *p*: *p*-value of the difference in percentage. [a] N= 299 with CHD, 5809 without CHD; [b] N= 315 with CHD, 6174 without CHD; [c] N= 213 with CHD, 2691 without CHD.

AIP: atherogenic index of plasma; BPLT: blood pressure-lowering drug therapy; BRI: body roundness index; BSI: body shape index; CKD: chronic kidney disease; CUN-BAE: according to its acronym in Spanish, *Clínica Universitaria de Navarra* - Body Adiposity Estimator; eGFR: estimated glomerular filtration rate; GLT: glycaemic-lowering drug therapy; HDL-c: high-density lipoprotein cholesterol; LAP: lipid accumulation product index; LLT: lipid-lowering drug therapy; TyG: triglyceride-glucose index; VAI: visceral adiposity index. The definitions of diseases or medical conditions are shown in Table S1 (Supplementary Materials).

Table S8. Diseases and medical conditions in populations with and without Stroke

	**With stroke** N= 250 **No. (%)**	**Without stroke** N= 6338 **No. (%)**	**With stroke *vs.* without stroke**	
			p	**OR** (95% CI)
Male	128 (51.2)	2776 (43.8)	0.021	1.3 (1.0–1.7)
Current smoking	38 (15.2)	1388 (21.9)	0.012	0.6 (0.5–0.9)
Alcoholism	18 (7.2)	592 (9.3)	0.252	0.8 (0.5–1.2)
Physical inactivity	137 (54.8)	2942 (46.4)	0.009	1.4 (1.1–1.8)
Overweight	95 (38.0)	2421 (38.2)	0.950	1.0 (0.8–1.3)
Obesity	101 (40.4)	1732 (27.3)	< 0.001	1.8 (1.4–2.3)
Abdominal obesity	149 (59.6)	2773 (43.8)	< 0.001	1.9 (1.5–2.5)
High-WtHR	141 (56.4)	2220 (35.0)	< 0.001	2.4 (1.9–3.1)
CUN-BAE excess adiposity	234 (93.6)	4598 (72.5)	< 0.001	5.5 (3.3–9.2)
High-VAI	55 (22.0)	1061 (16.7)	0.030	1.4 (1.0–1.9)
High-BSI	114 (45.6)	2055 (32.4)	< 0.001	1.7 (1.4–2.3)
High-BRI	130 (52.0)	1824 (28.8)	< 0.001	2.7 (2.1–3.5)
High-LAP index	89 (35.6)	1722 (27.2)	0.003	1.5 (1.1–1.9)
Prediabetes	77 (30.8)	1372 (21.6)	0.001	1.6 (1.2–2.1)
Diabetes	89 (35.6)	947 (14.9)	< 0.001	3.1 (2.4–4.1)
Hypertension	208 (83.2)	2478 (39.1)	< 0.001	7.7 (5.5–10.8)
Hypercholesterolaemia	218 (87.2)	3914 (61.8)	< 0.001	4.2 (2.9–6.1)
Low HDL-c	93 (37.2)	1726 (27.2)	0.001	1.6 (1.2–2.1)
Hypertriglyceridaemia	99 (39.6)	1848 (29.2)	< 0.001	1.6 (1.2–2.1)
Atherogenic dyslipidaemia	53 (21.2)	888 (14.0)	0.001	1.7 (1.2–2.3)
High-AIP	44 (17.6)	932 (14.7)	0.206	1.2 (0.9–1.7)
High-TyG index	98 (39.2)	1974 (31.1)	0.007	1.4 (1.1–1.8)
Metabolic syndrome	202 (80.8)	2649 (41.8)	< 0.001	5.9 (4.3–8.1)
Fatty liver index ≥ 60 [a]	113 (47.7)	2037 (34.7)	< 0.001	1.7 (1.3–2.2)
Hyperuricaemia [b]	46 (18.6)	694 (11.1)	< 0.001	1.8 (1.3–2.5)
Coronary heart disease	48 (19.2)	273 (4.3)	< 0.001	5.3 (3.8–7.4)
Peripheral arterial disease	34 (13.6)	116 (1.8)	< 0.001	8.4 (5.6–12.7)
Erectile dysfunction [c]	71 (55.5)	433 (15.6)	< 0.001	6.7 (4.7–9.7)
Heart failure	40 (16.0)	144 (2.3)	< 0.001	8.2 (5.6–11.9)
Atrial fibrillation	47 (18.8)	203 (3.2)	< 0.001	7.0 (4.9–9.9)
Albuminuria	44 (17.6)	350 (5.5)	< 0.001	3.7 (2.6–5.1)
Low eGFR	65 (26.0)	459 (7.2)	< 0.001	4.5 (3.3–6.1)
CKD	82 (32.8)	674 (10.6)	< 0.001	4.1 (3.1–5.4)
CKD low risk	168 (67.2)	5664 (89.4)	< 0.001	0.2 (0.2–0.3)
CKD moderate risk	43 (17.2)	447 (7.1)	< 0.001	2.7 (1.9–3.9)
CKD high risk	21 (8.4)	143 (2.3)	< 0.001	4.0 (2.5–6.4)
CKD very high risk	18 (7.2)	84 (1.3)	< 0.001	5.8 (3.4–9.8)
LLT	176 (70.4)	1676 (26.4)	< 0.001	6.6 (5.0–8.7)
BPLT	188 (75.2)	2151 (33.9)	< 0.001	5.9 (4.4–7.9)
GLT	75 (30.0)	772 (12.2)	< 0.001	3.1 (2.3–4.1)

No. (%): cases number (percentage); CI: confidence interval; OR: odds ratio; *p*: *p*-value of the difference in percentage. [a] N= 237 with stroke, 5871 without stroke; [b] N= 247 with stroke, 6242 without stroke; [c] N= 128 with stroke, 2776 without stroke.

AIP: atherogenic index of plasma; BPLT: blood pressure-lowering drug therapy; BRI: body roundness index; BSI: body shape index; CKD: chronic kidney disease; CUN-BAE: according to its acronym in Spanish, *Clínica Universitaria de Navarra* - Body Adiposity Estimator; eGFR: estimated glomerular filtration rate; GLT: glycaemic-lowering drug therapy; HDL-c: high-density lipoprotein cholesterol; LAP: lipid accumulation product index; LLT: lipid-lowering drug therapy; TyG: triglyceride-glucose index; VAI: visceral adiposity index; WtHR: waist-to-height ratio. The definitions of diseases or medical conditions are shown in Table S1 (Supplementary Materials).

Table S9. Diseases and medical conditions in populations with and without PAD

	**With PAD** N= 150 **No. (%)**	**Without PAD** N= 6438 **No. (%)**	**With PAD *vs.* without PAD**	
			p	**OR** (95% CI)
Male	94 (62.7)	2810 (43.6)	< 0.001	2.2 (1.6–3.0)
Current smoking	29 (19.3)	1397 (21.7)	0.487	0.9 (0.6–1.3)
Alcoholism	20 (13.3)	590 (9.2)	0.082	1.5 (1.0–2.5)
Physical inactivity	76 (50.7)	3003 (46.6)	0.329	1.2 (0.8–1.6)
Overweight	62 (41.3)	2454 (38.1)	0.423	1.1 (0.8–1.6)
Obesity	58 (38.7)	1775 (27.6)	0.003	1.7 (1.2–2.3)
Abdominal obesity	84 (56.0)	2838 (44.1)	0.004	1.6 (1.2–2.2)
High-WtHR	82 (54.7)	2279 (35.4)	< 0.001	2.2 (1.6–3.0)
CUN-BAE excess adiposity	138 (92.0)	4694 (72.9)	< 0.001	4.3 (2.4–7.7)
High-VAI	30 (20.0)	1086 (16.9)	0.312	1.2 (0.8–1.8)
High-BSI	68 (45.3)	2101 (32.6)	0.001	1.7 (1.2–2.4)
High-BRI	68 (45.3)	1886 (29.3)	< 0.001	2.0 (1.4–2.8)
High-LAP index	56 (37.3)	1755 (27.3)	0.006	1.6 (1.1–2.2)
Prediabetes	35 (23.3)	1414 (22.0)	0.689	1.1 (0.7–1.6)
Diabetes	72 (48.0)	964 (15.0)	< 0.001	5.2 (3.8–7.3)
Hypertension	131 (87.3)	2555 (39.7)	< 0.001	10.5 (6.5–17.0)
Hypercholesterolaemia	123 (82.0)	4009 (62.3)	< 0.001	2.8 (1.8–4.2)
Low HDL-c	61 (40.7)	1758 (27.3)	< 0.001	1.8 (1.3–2.5)
Hypertriglyceridaemia	68 (45.3)	1879 (29.2)	< 0.001	2.0 (1.5–2.8)
Atherogenic dyslipidaemia	38 (25.3)	903 (14.0)	< 0.001	2.1 (1.4–3.0)
High-AIP	27 (18.0)	949 (14.7)	0.267	1.3 (0.8–1.9)
High-TyG index	65 (43.3)	2007 (31.2)	0.002	1.7 (1.2–2.3)
Metabolic syndrome	127 (84.7)	2724 (42.3)	< 0.001	7.5 (4.8–11.8)
Fatty liver index ≥ 60 [a]	75 (52.1)	2075 (34.8)	< 0.001	2.0 (1.5–2.8)
Hyperuricaemia [b]	33 (22.3)	707 (11.1)	< 0.001	2.3 (1.5–3.4)
Coronary heart disease	37 (24.7)	284 (4.4)	< 0.001	7.1 (4.8–10.5)
Stroke	34 (22.7)	216 (3.4)	< 0.001	8.4 (5.6–12.7)
Erectile dysfunction [c]	69 (73.4)	435 (15.5)	< 0.001	15.1 (9.4–24.1)
Heart failure	25 (16.7)	159 (2.5)	< 0.001	7.9 (5.0–12.5)
Atrial fibrillation	22 (14.7)	228 (3.5)	< 0.001	4.7 (2.9–7.5)
Albuminuria	35 (23.3)	359 (5.6)	< 0.001	5.2 (3.5–7.6)
Low eGFR	48 (32.0)	476 (7.4)	< 0.001	5.9 (4.1–8.4)
CKD	61 (40.7)	695 (10.8)	< 0.001	5.7 (4.1–7.9)
CKD low risk	89 (59.3)	5743 (89.2)	< 0.001	0.2 (0.1–0.2)
CKD moderate risk	26 (17.3)	464 (7.2)	< 0.001	2.7 (1.8–4.2)
CKD high risk	21 (14.0)	143 (2.2)	< 0.001	7.2 (4.4–11.7)
CKD very high risk	14 (9.3)	88 (1.4)	< 0.001	7.4 (4.1–13.4)
LLT	107 (71.3)	1745 (27.1)	< 0.001	6.7 (4.7–9.6)
BPLT	119 (79.3)	2220 (34.5)	< 0.001	7.3 (4.9–10.9)
GLT	55 (36.7)	792 (12.3)	< 0.001	4.1 (2.9–5.8)

PAD: peripheral arterial disease; No. (%): cases number (percentage); CI: confidence interval; OR: odds ratio; *p*: *p*-value of the difference in percentage. [a] N= 144 with PAD, 5964 without PAD; [b] N= 148 with PAD, 6341 without PAD; [c] N= 94 with PAD, 2810 without PAD.

AIP: atherogenic index of plasma; BPLT: blood pressure-lowering drug therapy; BRI: body roundness index; BSI: body shape index;; CKD: chronic kidney disease; CUN-BAE: according to its acronym in Spanish, *Clínica Universitaria de Navarra* - Body Adiposity Estimator; eGFR: estimated glomerular filtration rate; GLT: glycaemic-lowering drug therapy; HDL-c: high-density lipoprotein cholesterol; LAP: lipid accumulation product index; LLT: lipid-lowering drug therapy; TyG: triglyceride-glucose index; VAI: visceral adiposity index; WtHR: waist-to-height ratio. The definitions of diseases or medical conditions are shown in Table S1 (Supplementary Materials).

Table S10. Diseases and medical conditions in populations with and without ASCVD

	**With ASCVD** N= 615 **No. (%)**	**Without ASCVD** N= 5973 **No. (%)**	**With *vs.* without ASCVD**	
			p	**OR** (95% CI)
Male	372 (60.5)	2532 (42.4)	< 0.001	2.1 (1.8–2.5)
Current smoking	97 (15.8)	1329 (22.3)	0.061	0.7 (0.5–0.8)
Alcoholism	58 (9.4)	552 (9.2)	0.877	1.0 (0.8–1.4)
Physical inactivity	308 (50.1)	2771 (46.4)	0.081	1.2 (1.0–1.4)
Overweight	244 (39.7)	2272 (38.0)	0.426	1.1 (0.9–1.3)
Obesity	245 (39.8)	1588 (26.6)	< 0.001	1.8 (1.5–2.2)
Abdominal obesity	355 (57.7)	2567 (43.0)	< 0.001	1.8 (1.5–2.1)
High-WtHR	348 (56.6)	2013 (33.7)	< 0.001	2.6 (2.2–3.0)
CUN-BAE excess adiposity	573 (93.2)	4259 (71.3)	< 0.001	5.5 (4.0–7.5)
High-VAI	131 (21.3)	985 (16.5)	0.002	1.4 (1.1–1.7)
High-BSI	284 (46.2)	1885 (31.6)	< 0.001	1.9 (1.6–2.2)
High-BRI	305 (49.6)	1649 (27.6)	< 0.001	2.6 (2.2–3.1)
High-LAP index	228 (37.1)	1583 (26.5)	< 0.001	1.6 (1.4–1.9)
Prediabetes	182 (29.6)	1267 (21.2)	< 0.001	1.6 (1.3–1.9)
Diabetes	239 (38.9)	797 (13.3)	< 0.001	4.1 (3.5–4.9)
Hypertension	535 (87.0)	2151 (36.0)	< 0.001	11.9 (9.3–15.1)
Hypercholesterolaemia	537 (87.3)	3595 (60.2)	< 0.001	4.6 (3.6–5.8)
Low HDL-c	239 (38.9)	1580 (26.5)	< 0.001	1.8 (1.5–2.1)
Hypertriglyceridaemia	250 (40.7)	1697 (28.4)	< 0.001	1.7 (1.5–2.0)
Atherogenic dyslipidaemia	136 (22.1)	805 (13.5)	< 0.001	1.8 (1.5–2.2)
High-AIP	110 (17.9)	866 (14.5)	0.024	1.3 (1.0–1.6)
High-TyG index	263 (42.8)	1809 (30.3)	< 0.001	1.7 (1.5–2.0)
Metabolic syndrome	526 (85.5)	2325 (38.9)	< 0.001	9.3 (7.4–11.7)
Fatty liver index ≥ 60 [a]	290 (50.0)	1860 (33.6)	< 0.001	2.0 (1.9–2.3)
Hyperuricaemia [b]	117 (19.3)	623 (10.6)	< 0.001	2.0 (1.6–2.5)
Erectile dysfunction [c]	215 (57.8)	289 (11.4)	< 0.001	10.6 (8.4–13.5)
Heart failure	87 (14.1)	97 (1.6)	< 0.001	10.0 (7.4–13.5)
Atrial fibrillation	84 (13.7)	166 (2.8)	< 0.001	5.5 (4.2–7.3)
Albuminuria	102 (16.6)	292 (4.9)	< 0.001	3.9 (3.0–4.9)
Low eGFR	155 (25.2)	369 (6.2)	< 0.001	5.1 (4.1–6.3)
CKD	196 (31.9)	560 (9.4)	< 0.001	4.5 (3.7–5.5)
CKD low risk	419 (68.1)	5413 (90.6)	< 0.001	0.2 (0.2–0.3)
CKD moderate risk	102 (16.6)	388 (6.5)	< 0.001	2.9 (2.3–3.6)
CKD high risk	54 (8.8)	110 (1.8)	< 0.001	5.1 (3.7–7.2)
CKD very high risk	40 (6.5)	62 (1.0)	< 0.001	6.6 (4.4–10.0)
LLT	461 (75.0)	1391 (23.3)	< 0.001	9.9 (8.1–11.9)
BPLT	501 (81.5)	1838 (30.8)	< 0.001	9.9 (8.0–12.2)
GLT	195 (31.7)	652 (10.9)	< 0.001	3.8 (3.1–4.6)

ASCVD: atherosclerotic cardiovascular disease; No. (%): cases number (percentage); CI: confidence interval; OR: odds ratio; *p*: *p*-value of the difference in percentage. [a] N= 580 with ASCVD, 5528 without ASCVD; [b] N= 605 with ASCVD, 5884 without ASCVD; [c] N= 372 with ASCVD, 2532 without ASCVD.

AIP: atherogenic index of plasma; BPLT: blood pressure-lowering drug therapy; BRI: body roundness index; BSI: body shape index; CKD: chronic kidney disease; CUN-BAE: according to its acronym in Spanish, *Clínica Universitaria de Navarra* - Body Adiposity Estimator; eGFR: estimated glomerular filtration rate; GLT: glycaemic-lowering drug therapy; HDL-c: high-density lipoprotein cholesterol; LAP: lipid accumulation product index; LLT: lipid-lowering drug therapy; TyG: triglyceride-glucose index; VAI: visceral adiposity index; WtHR: waist-to-height ratio. The definitions of diseases or medical conditions are shown in Table S1 (Supplementary Materials).

Table S11. Multivariate analysis of diseases and medical conditions for CHD **(a),** for Stroke **(b)**, for PAD **(c)**, and for ASCVD **(d)**

	Wald	β [a]	OR Exp(β) [b]	*p* [c]
Hypertension	105.5	2.38 (0.23)	10.77 (6.84–16.95)	< 0.001
Hypercholesterolaemia	37.5	1.33 (0.22)	3.78 (2.47–5.77)	< 0.001
Heart failure	38.9	1.18 (0.19)	3.26 (2.25–4.73)	< 0.001
Diabetes	20.4	0.69 (0.15)	1.99 (1.48–2.69)	< 0.001
Low eGFR	11.5	0.51 (0.15)	1.66 (1.24–2.23)	0.001
Low HDL-c	8.1	0.36 (0.13)	1.43 (1.12–1.84)	0.004
Prediabetes	5.8	0.38 (0.16)	1.47 (1.07–2.00)	0.016

(a)

	Wald	β [a]	OR Exp(β) [b]	*p* [c]
Hypertension	59.6	1.43 (0.19)	4.20 (2.20–6.04)	< 0.001
Atrial fibrillation	21.3	0.94 (0.21)	2.57 (1.72–3.84)	< 0.001
Heart failure	17.9	0.94 (0.21)	2.55 (1.65–3.94)	< 0.001
Hypercholesterolaemia	20.3	0.90 (0.20)	2.45 (1.66–3.61)	< 0.001
Diabetes	12.2	0.59 (0.17)	1.80 (1.29–2.51)	< 0.001
Prediabetes	6.6	0.43 (0.17)	1.54 (1.11–2.14)	0.010

(b)

	Wald	β [a]	OR Exp(β) [b]	*p* [c]
Hypertension	55.3	1.92 (0.24)	6.82 (4.11–11.31)	< 0.001
Heart failure	24.1	1.18 (0.24)	3.26 (2.03–5.22)	< 0.001
Diabetes	30.3	0.97 (0.18)	2.63 (1.86–3.71)	< 0.001

(c)

	Wald	β [a]	OR Exp(β) [b]	*p* [c]
Hypertension	186.0	1.80 (0.13)	6.05 (4.67–7.84)	< 0.001
Heart failure	34.9	1.06 (0.18)	2.90 (2.04–4.12)	< 0.001
Hypercholesterolaemia	47.7	0.92 (0.13)	2.50 (1.93–3.24)	< 0.001
Diabetes	41.0	0.75 (0.12)	2.11 (1.68–2.65)	< 0.001
Low eGFR	24.1	0.59 (0.12)	1.81 (1.43–2.29)	< 0.001
Atrial fibrillation	6.7	0.44 (0.17)	1.55 (1.11–2.15)	0.010
Prediabetes	13.6	0.43 (0.12)	1.54 (1.22–1.93)	< 0.001
Low HDL-c	5.8	0.24 (0.10)	1.27 (1.05–1.54)	0.016

(d)

[a] β coefficient (± deviation); [b] OR Exp (β): odds-ratio (95% confidence interval); [c] *p*: *p*-value of Wald test with one degree of freedom. ASCVD: atherosclerotic cardiovascular disease; CHD: coronary heart disease; eGFR: estimated glomerular filtration rate; HDL-c: high-density lipoprotein cholesterol; PAD: peripheral arterial disease. Definitions of the diseases and clinical conditions are shown in Table S1 (Supplementary Materials).
